# Loss of O-GlcNAcylation in cardiac myocytes triggers the integrated stress response, contributing to heart failure

**DOI:** 10.1016/j.jbc.2025.110818

**Published:** 2025-10-14

**Authors:** Kyriakos N. Papanicolaou, Wenxi Zhang, Aidan J. Dunphy, Justin C. Zhong, Clara Y. Cho, Dan R. Turner, Deepthi Ashok, D. Brian Foster, Brian O’Rourke, Natasha E. Zachara

**Affiliations:** 1 Division of Cardiology, Department of Medicine, The Johns Hopkins University School of Medicine, Baltimore, Maryland, USA; 2 Department of Biophysics and Biophysical Chemistry, The Johns Hopkins University School of Medicine, Baltimore, Maryland, USA; 3 Department of Oncology, The Johns Hopkins University School of Medicine, Baltimore, Maryland, USA

**Keywords:** OGT, GCN2, PERK, mTOR, eIF2α, Atf4, translation initiation, ISRIB, cardiomyocytes, cardiomyopathy

## Abstract

Heart failure (HF) is a significant global health problem, affecting an estimated 64 million people worldwide. At the core of HF is the progressive dysfunction and irreversible loss of cardiac myocytes. O-GlcNAc transferase (OGT) is a conserved enzyme that catalyzes the addition of N-acetyl-glucosamine (GlcNAc) to serine or threonine residues of intracellular proteins. This dynamic protein modification, termed O-GlcNAcylation, has been implicated in nutrient sensing, metabolic regulation and stress adaptation. The integrated stress response (ISR) is a pathway that enables cells to rapidly respond to acute environmental changes and cell damage. During ISR, the translation factor eIF2α is phosphorylated, shutting down general translation but favoring the rapid production of stress-adaptive proteins. However, prolonged activation of the ISR can be detrimental to cells. In this study, we found that inhibiting OGT activates the GCN2/eIF2α/Atf4 signaling axis of the ISR. Activation of this pathway could be blocked by ISRIB, a small molecule that opposes the activity of phosphorylated eIF2α. Mice with inducible deletion of OGT in adult cardiomyocytes developed HF, and treatment with ISRIB significantly delayed the progression to HF. Our study reveals the regulatory impact of O-GlcNAcylation on the ISR and highlights a new potential strategy for alleviating HF.

Heart failure (HF) is a leading cause of morbidity and mortality, affecting over 64 million people worldwide, with greater than 50% mortality rate within the first 5 years of diagnosis (1). Current drugs, such as ACE inhibitors, beta blockers, and SGLT2 inhibitors have proven to be beneficial for the treatment of HF. However, the burden of the disease is projected to keep rising; in the United States for example, it is expected to affect more than eight million people by 2030 (2). This surge in numbers necessitates a better understanding of the underlying molecular causes of the disease so that better treatment strategies can be developed for HF.

O-GlcNAcylation is the reversible modification of intracellular proteins by the monosaccharide O-GlcNAc (O-linked N-acetyl glucosamine). The enzyme O-GlcNAc transferase (OGT) uses uridine diphosphate N-acetylglucosamine (UDP-GlcNAc) as the high-energy sugar donor to covalently link the GlcNAc moiety to Ser/Thr residues on thousands of intracellular proteins, while the removal of O-GlcNAc from proteins is catalyzed by O-GlcNAcase (OGA) (3, 4, 5, 6). O-GlcNAcylation is highly dynamic and plays important roles in nutrient sensing and stress response pathways (7, 8, 9, 10, 11, 12). Moreover, O-GlcNAcylation has emerged as an important factor in chronic disease (13, 14), including cardiac conditions such as hypertrophic dysfunction (15, 16, 17, 18), diabetic arrhythmias and cardiomyopathy (19, 20, 21), and heart failure (22, 23, 24). The current view suggests that transient elevation of O-GlcNAcylation serves a protective role against injury, whereas prolonged increases or decreases exacerbate cardiac pathology (25, 26, 27, 28, 29). Finally, O-GlcNAcylation in hearts and cardiac myocytes has been investigated in relation to phosphorylation pathways, where it appears to be important for the crosstalk with stress kinases such as the Ca^2+^/calmodulin-dependent kinase CaMKII, the AMP-dependent protein kinase (AMPK), the growth regulator mTOR, and the mitogen-activated protein kinases (MAPKs) p38 and Erk1/2 (30, 31, 32, 33).

The integrated stress response (ISR) is regulated by four upstream kinases that, upon activation phosphorylate eIF2α, a subunit of the translation initiation factor eIF2. The four kinases are HRI, PKR, PERK and GCN2 (EIF2AK1, EIF2AK2, EIF2AK3 and EIF2AK4, respectively). Each kinase is activated by one or more cellular stresses, including a shortage of nutrients, accumulation of unfolded proteins, viral replication, hypoxia, mitochondrial dysfunction, or reactive oxygen species production (34, 35, 36, 37, 38, 39). Phosphorylation of eIF2α is critical in regulating the mRNAs to be translated, as it slows down the translation of mRNAs with 5′-7′ methyl guanosine cap and favors translation of mRNAs that have short upstream open reading frames (uORFs) (40, 41). As a result, when eIF2α is phosphorylated, general translation wanes. In contrast, the translation of mRNAs with uORFs is prioritized, leading to the production of proteins important in stress adaptation (42). The transcription factor Atf4 is one of the first proteins to accumulate during the ISR, which then promotes the expression of antioxidant genes, amino acid transporters, metabolic genes important in stress adaptation, autophagy genes, and cell death mediators (35, 43, 44, 45). While the ISR evolved to confer protection against acute stress, emerging evidence suggests that chronic low-level activation of the ISR is implicated in neurological and other disorders (46, 47, 48, 49, 50, 51, 52, 53).

In cardiomyocytes and the heart, pharmacologic enhancement of eIF2α phosphorylation is protective in myocardial infarction (54, 55), although in other reports, antagonizing eIF2α phosphorylation upon reperfusion appears to be beneficial (56, 57). Persistent activation of the PERK/eIF2α axis causes cardiomyocyte atrophy (58), whereas acute activation may be protective in ischemia/reperfusion injury (59). Thus, it appears that a well-controlled and precisely regulated ISR can be beneficial for the heart, offering opportunities for new therapeutic approaches (60, 61).

In this work, we found that short-term inhibition of OGT in neonatal cardiomyocytes induces the phosphorylation of eIF2α concomitant with a reduction in global protein synthesis and accumulation of stress adaptive transcription factors Atf4 and Chop. We show that the nutrient sensor GCN2 plays an important role, initiating a GCN2/eIF2α/Atf4 signaling axis. Supporting this model, the ISR inhibitor ISRIB counteracted the accumulation of Atf4 caused by OGT inhibition. Mice with conditional inactivation of OGT in adult cardiomyocytes developed HF and had elevated GCN2 and eIF2α protein levels. Chronic infusion with ISRIB significantly delayed the progression of cardiac dysfunction in OGT-deficient mice. Our data reveal, for the first time, an important relationship between O-GlcNAcylation and the ISR in cardiac myocytes and highlights the potential of targeting the ISR as a new therapeutic strategy in cardiac pathophysiology, including dilated cardiomyopathy and heart failure.

## Results

### OGT inhibition suppresses nascent protein synthesis and induces phosphorylation of the translational regulator eIF2α in cardiac myocytes

**Figure 1:**
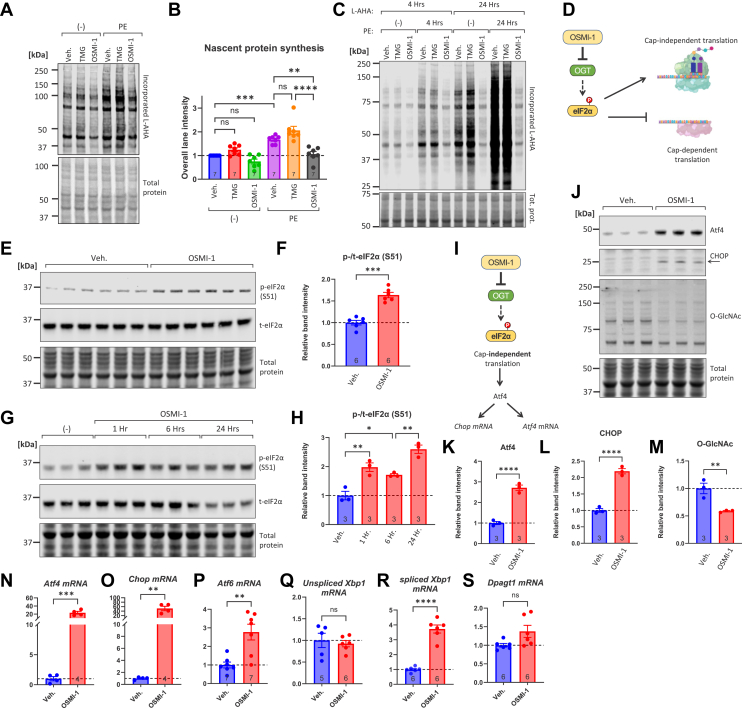
**OGT inhibition in cardiac myocytes suppresses nascent protein synthesis and induces phosphorylation of the translational regulator eIF2α.***A–B*, primary neonatal rat ventricular myocytes (NRVMs) were incubated in serum-free medium for 24 h, then treated with Vehicle (0.1% DMSO), OGA inhibitor TMG (200 nM) or OGT inhibitor OSMI-1 (25 μM) for 6 h. Cells were exposed to phenylephrine (PE, 5 μM) in medium with L-azidohomoalanine (L-AHA, 25 μM) replacing L-methionine. After 4 h, cells were harvested and L-AHA reacted with biotin-alkyne. Streptavidin immunoblot band intensities were used to measure nascent protein synthesis. *C*, representative streptavidin blot of L-AHA incorporation into nascent proteins in NRVMs stimulated for four or 24 h with PE (5 μM) in the presence of Vehicle (0.1% DMSO), TMG or OSMI-1. *D*, schematic depicting eIF2α phosphorylation's effect on cap-independent translation and OGT inhibition's putative effect on eIF2α phosphorylation. *E–F*, Western blot analysis of phospho- and total eIF2α following OSMI-1 treatment (25 μM, 6 h). *G–H*, Western blot analysis and quantitation of eIF2α and S6 phosphorylation following OSMI-1 treatment (25 μM) for 1, 6, and 24 h. *I*, schematic illustrating the proposed mechanism: OSMI-1 inhibits OGT, leading to reduced protein O-GlcNAcylation. This reduction is associated with increased phosphorylation of eIF2α. Phosphorylated eIF2α selectively enhances the translation of Activating Transcription Factor 4 (ATF4). Elevated ATF4 protein levels then promote the transcription of ATF4 and C/EBP Homologous Protein (CHOP) genes, resulting in increased mRNA and protein levels of both ATF4 and CHOP. *J–M*, Western blot analysis of Atf4, CHOP, and O-GlcNAc following OSMI-1 treatment (25 μM, 6 h). *N–S*, qPCR analysis of mRNAs in DMSO- or OSMI-1-treated cells (25 μM, 24 h). Group sizes shown in respective bars. Statistical Analysis: Bars show mean ± standard error of the mean (SEM); dots denote individual biological replicates; numerals on bars indicate sample size (these conventions apply to all figures). Comparisons between vehicle (DMSO) and OSMI-1-treated samples in (*F*, *K*–*S*) used unpaired Student's *t* test. ns: not significant (*p* > 0.05), ∗*p* < 0.05, ∗∗*p* < 0.01, ∗∗∗*p* < 0.001, ∗∗∗∗*p* < 0.0001. Comparisons across treatment groups in B and H used one-way ANOVA followed by Tukey's *post hoc* test to identify differences between specific groups. Complete ANOVA statistics are reported in Table S4.

Previously, we demonstrated that OGT inhibition impairs the hypertrophic response of neonatal rat ventricular cardiomyocytes (NRVMs) to phenylephrine (PE) (33). Physiologic hypertrophy of cardiomyocytes involves the acceleration of translation rates and the rapid production of new polypeptides (62). To investigate whether the blockade in hypertrophic response was accompanied by reductions in nascent protein synthesis, we treated NRVMs with the OGT inhibitor OSMI-1 or vehicle, with or without PE. PE alone (5 μM, 4 h) significantly increased L-AHA incorporation, a direct measure of nascent protein synthesis (Fig. 1, *A* and *B*, compare blue with purple bars). However, treatment with the OGT-specific inhibitor OSMI-1 (25 μM, 6 h before and 4 h after PE) nearly completely blocked PE-stimulated L-AHA incorporation (Fig. 1, *A* and *B*). The translation-inhibiting effect of OSMI-1 was evident both at 4 and at 24 h of PE exposure (Fig. 1*C*). A key regulator of translation rates is eIF2α (encoded by the gene *EIF2S1*), whose phosphorylation at Ser 51 during stress halts protein translation (41). We therefore examined whether OGT inhibition by OSMI-1 altered the phosphorylation of eIF2α (Fig. 1*D*). Indeed, we found that treatment with OSMI-1 (25 μM, 6 h) strongly increased phospho-eIF2α, while the levels of total eIF2α remained constant (Fig. 1, *E* and *F*). These findings were further bolstered by time course experiments, which showed that p-eIF2α increases at 1 h and remains high at 6 h of OSMI-1 treatment (Fig. 1, *G* and *H*). Interestingly, we found that the levels of total eIF2α remained constant at 1 and 6 h but were decreased at 24 h of OSMI-1 treatment (Fig. 1*G*, total eIF2α blot).

It had been previously reported that O-GlcNAcylation directly modifies the proteasome and negatively regulates its proteolytic function (63, 64); therefore, a reduction in O-GlcNAcylation could impact overall protein levels through overactivation of the proteasome. While the L-AHA incorporation assay monitors direct changes in translation rates (65), we used this to address any effects of OSMI-1 on protein turnover. Treating cells with the proteasome inhibitor MG132 (500 nM, or 1 μM for 6 h) did not rescue the reduction in nascent polypeptides induced by OSMI-1 (Fig. S1*A*). Interestingly, OSMI-1 appeared to increase the abundance of ubiquitinated proteins to levels similar to those observed when MG132 was used (Fig. S1*B*). Finally, MG132 did not alter the effect of eIF2α phosphorylation, or the reduction in O-GlcNAcylation caused by OSMI-1 (Fig. S1, *C*–*E*).

To verify the contribution of O-GlcNAc cycling to eIF2α phosphorylation, cells were first treated with the OGA inhibitor Thiamet-G (TMG, 3 h) to elevate O-GlcNAc levels, followed by OGT inhibition with OSMI-1 (3 h). TMG alone did not alter p-eIF2α levels, but it significantly decreased the induction caused by OSMI-1 (Fig. S2, *A* and *B*). As expected, treatment with TMG significantly elevated global O-GlcNAcylation (∼94% increase relative to untreated controls, Fig. S2, *C* and *D*). Importantly, this elevation was still evident but reduced when OSMI-1 was added after 3 h of TMG pretreatment (∼52% increase over baseline). In contrast, OSMI-1 treatment alone for 3 h did not produce a detectable reduction in global O-GlcNAcylation, consistent with the relatively slow turnover of many O-GlcNAc-modified proteins. Taken together, these results suggest that OSMI-1–induced eIF2α phosphorylation can be alleviated by reciprocal increases in O-GlcNAcylation, indicating that this molecular event is associated with dynamic changes in O-GlcNAcylation.

To further establish that OGT inhibition leads to eIF2α phosphorylation, we tested additional OGT inhibitors, including OSMI-2, which is structurally related to OSMI-1 (66), and 5S-GlcNHex, which is structurally distinct (67). In both cases, OGT inhibition was accompanied by a significant increase in eIF2α phosphorylation, resembling the effect observed with OSMI-1 (Fig. S2, *E* and *F*). As expected, both OSMI-2 and 5S-GlcNHex significantly reduced global O-GlcNAcylation (55% and 41%, respectively, Fig. S2, *G* and *H*). Collectively, these results point to a consistent biological effect where inhibiting OGT activity induces eIF2α phosphorylation.

Phosphorylation of eIF2α is a pivotal event in the integrated stress response (ISR), slowing cap-dependent translation while favoring the translation of cap-independent mRNAs, like Atf4 (Fig. 1*I*). Consistent with activation of the ISR, we observed a strong increase in both Atf4 and Chop proteins after 6 h of exposure to OSMI-1 (Fig. 1, *J*–*M*). Furthermore, the levels of O-GlcNAc were decreased by 50% (Fig. 1, *J* and *M*), consistent with our previous observations (33). To corroborate these findings, we examined the mRNA levels of Atf4 and Chop, which were robustly increased after 24 h of OSMI-1 treatment (Fig. 1, *N* and *O*). The endoplasmic reticulum (ER) stress-inducible transcription factors Atf6 and spliced Xbp1 (Xbp1s) were also increased (Fig. 1, *P*–*R*), while the expression of the key enzyme for co-translational N-linked glycosylation of ER-resident proteins Dpagt1 was not changed (Fig. 1*S*).

**Figure 2:**
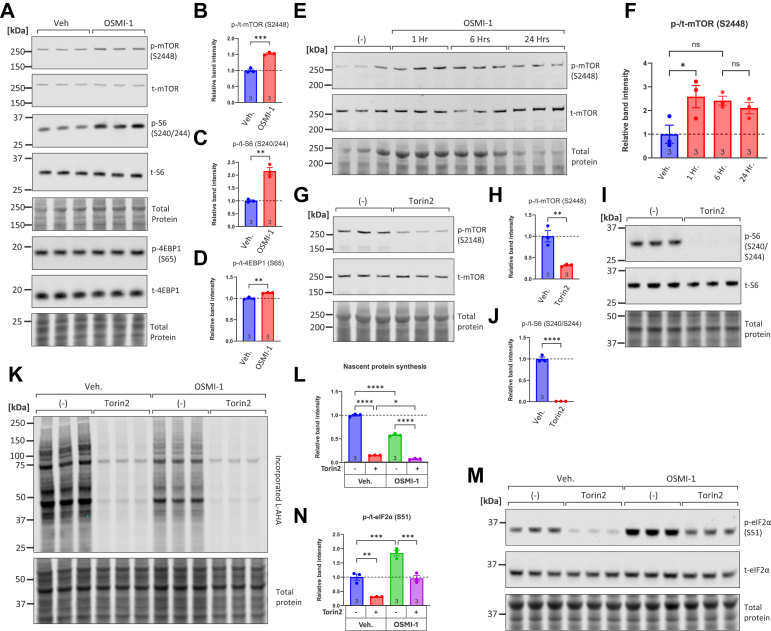
**OGT inhibition activates mTOR phosphorylation and downstream signaling.***A–D*, NRVMs were exposed to OSMI-1 (25 μM, 6 h) and analyzed for phosphorylation of mTOR and its downstream targets S6 and 4EBP1 by Western blot. *E–F*, Western blot analysis and quantitation of phospho-mTOR and total mTOR following treatment with OSMI-1 (25 μM) for 1, 6, and 24 h. *G–J*, Western blot analysis of phospho-mTOR, total mTOR, phospho-S6, and total S6 in NRVM samples after treatment with Torin2 (1 μM). *K–L*, NRVMs were cultured in medium without serum for 24 h and then placed in L-AHA medium (DMEM depleted of L-methionine and L-cysteine and supplemented with 25 μM L-AHA) in groups containing the mTOR inhibitor Torin2 (1 μM) with or without OSMI-1 (25 μM). Six hours later, the cells were harvested and subjected to L-AHA-azide biotin-alkyne ‘click’ reaction, and the extent of L-AHA incorporation in each treatment group was assessed by streptavidin western blotting. *M-N*, Western blot analysis of phospho-eIF2α and total eIF2α after treatment for 6 h with OSMI-1 (25 μM), with or without Torin2 (1 μM). Bars represent means ± standard error. Comparisons between vehicle (DMSO) and OSMI-1-treated samples were performed using unpaired Student's *t* test. Comparisons among four groups were performed with one-way ANOVA followed by Tukey's *post hoc* test. ns: not significant, ∗*p* < 0.05, ∗∗*p* < 0.01, ∗∗∗*p* < 0.001, ∗∗∗∗*p* < 0.0001. Complete ANOVA statistics are reported in Table S4.

The mechanistic target of rapamycin (mTOR) is a well-known regulator of translation that is impacted by OGT and O-GlcNAcylation (68, 69). We therefore interrogated whether OGT inhibition affects the phosphorylation of mTOR and its downstream target rpS6 and 4EBP1. In contrast to our expectations, treatment of NRVMs with OSMI-1 increased the relative levels of phospho-mTOR and phospho-rpS6 and to a lesser extent p-4EBP1 (Fig. 2, *A* and *B*). Furthermore, a time course experiment revealed that the phosphorylation of mTOR peaks at 1 h after OSMI-1 treatment and gradually declines at 6 and 24 h afterwards (Fig. 2, *E* and *F*). We next examined whether mTOR influences NRVM translation rates. First, we demonstrated that Torin2, a selective mTOR inhibitor, could robustly decrease the baseline phosphorylation of mTOR and rpS6 (Fig. 2, *G*–*I*). Furthermore, treating NRVMs with Torin2 (1 μM, 6 h) significantly decreased L-AHA incorporation, to a degree that was higher than the one observed with OSMI-1 (Fig. 2, *K* and *L*). Finally, Torin2 was efficient at decreasing the baseline and OSMI-1-induced phosphorylation of eIF2α (Fig. 2, *M* and *N*).

Taken together, these findings suggest that OGT inhibition with OSMI-1 activates mTOR signaling. The fact that we find activation, instead of inhibition of mTOR signaling, indicates that the translational arrest is not due to loss of mTOR signaling. Interestingly, these studies show that mTOR may be implicated in the phosphorylation of eIF2α downstream of OGT inhibition.

### ISRIB prevents the activation of downstream ISR effector Atf4 but does not rescue the deficit in nascent polypeptide synthesis caused by OGT inhibition

**Figure 3:**
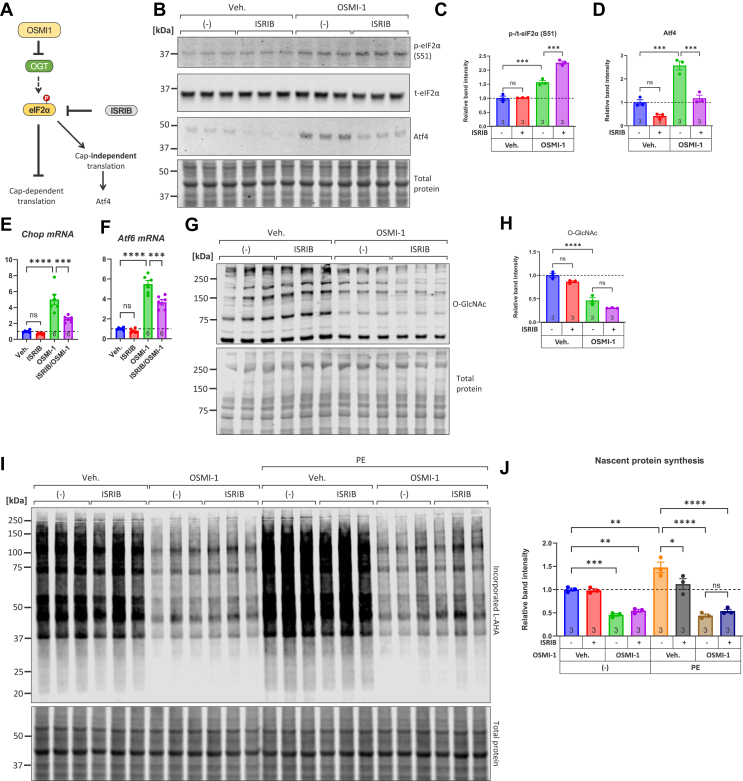
**ISRIB prevents the activation of downstream ISR effector Atf4 but does not rescue the deficit in nascent polypeptide synthesis caused by OGT inhibition.***A*, schematic depicting the proposed model: OSMI-1 inhibits OGT, leading to increased eIF2α phosphorylation and increases ATF4 protein levels through cap-independent translation. ISRIB, opposing the effects of phospho-eIF2α, is expected to reduce ATF4 translation and restore regular cap-dependent translation. *B–D*, Western blot analysis of phospho-eIF2α, total eIF2α, and ATF4 protein in NRVMs after treatment for 6 h with OSMI-1 (25 μM), with or without ISRIB (1 μM). *E–F*, qPCR analysis of Chop and Atf6 mRNAs in NRVMs treated for 24 h with or without OSMI-1 (25 μM) and with or without ISRIB (1 μM). *G–H*, Western blot and quantitation analysis of O-GlcNAc levels in NRVMs after treatment for 6 h with OSMI-1 (25 μM), with or without ISRIB (1 μM). *I–J*, NRVMs were cultured in medium without serum for 24 h and then placed in L-AHA medium (DMEM depleted of L-methionine and L-cysteine and supplemented with 25 μM L-AHA) in groups containing OSMI-1 (25 μM) and/or ISRIB (1 μM), with or without phenylephrine (PE, 5 μM). Six hours later, the cells were harvested and subjected to L-AHA-azide biotin-alkyne 'click' reaction, and the extent of L-AHA incorporation in each treatment group was assessed by streptavidin western blotting. Comparisons across groups were performed using one-way ANOVA with Tukey's *post hoc* test. ns: not significant, ∗*p* < 0.05, ∗∗*p* < 0.01, ∗∗∗*p* < 0.001, ∗∗∗∗*p* < 0.0001. Complete ANOVA statistics are reported in Table S4.

To corroborate the activation of the ISR by OGT inhibition in our experimental model, we tested whether the small molecule inhibitor of the ISR, ISRIB, could reverse the effects caused by OSMI-1 (Fig. 3*A*). ISRIB is known to inhibit the ISR by acting downstream of eIF2α phosphorylation (70, 71) and is therefore not a direct inhibitor of eIF2α phosphorylation. Consistently, we found that ISRIB did not reduce the basal levels of eIF2α but synergized with OGT inhibition to further elevate eIF2α phosphorylation (Fig. 3, *B* and *C*). As expected, ISRIB suppressed the accumulation of Atf4 caused by OGT inhibition (Fig. 3, *B* and *D*). We also examined the mRNA levels of Chop and Atf6, previously found to be upregulated by OSMI-1. Consistent with inhibition of the ISR, ISRIB significantly decreased the mRNA abundance of both factors, despite the presence of OSMI-1 (Fig. 3, *E* and *F*). ISRIB treatment had no significant effect on the decrease in O-GlcNAcylation induced by OSMI-1 (Fig. 3, *G* and *H*). Finally, we examined the potency of ISRIB to restore translational rates in OGT-inhibited NRVMs, in basal or PE-activated conditions. Consistent with previous findings, OSMI-1 strongly suppressed L-AHA incorporation both at baseline and in PE-stimulated conditions; however, co-treatment of the cells with ISRIB did not revert this suppression (Fig. 3, *I* and *J*). Interestingly, ISRIB alone appeared to counteract L-AHA incorporation caused by PE.

Taken together, these findings demonstrated that OGT inhibition by OSMI-1 induces the ISR, as several molecular markers of the ISR were reversed by the co-treatment with ISRIB. However, the translational impairment induced by OGT inhibition cannot be solely ascribed to activation of the ISR, suggesting that other mechanisms related to O-GlcNAcylation might be contributing to this effect.

### Inhibiting upstream eIF2α kinase Eif2ak4/GCN2 reduces eIF2α phosphorylation induced by OGT inhibition

**Figure 4:**
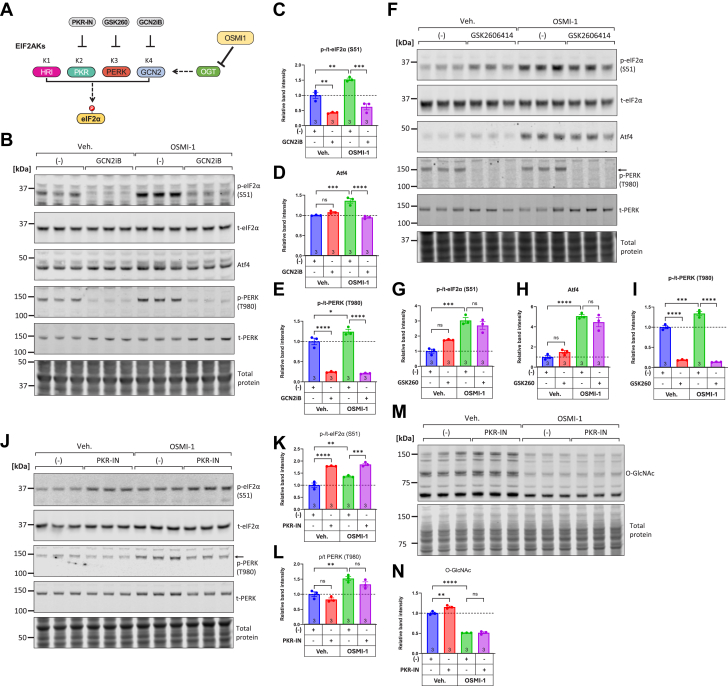
**Targeting upstream eIF2α kinases with small molecule inhibitors indicates the Eif2ak4/GCN2 is a key kinase responsible for eIF2α phosphorylation during OGT inhibition.***A*, schematic depicting the four eIF2α kinases: HRI (Heme-Regulated Inhibitor, EIF2AK1), PKR (Protein Kinase R, EIF2AK2), PERK (PKR-like ER Kinase, EIF2AK3), and GCN2 (General Control Non-derepressible 2, EIF2AK4), their respective small molecule inhibitors, and the potential impact of OGT inhibition by OSMI-1 in activating one or more of these kinases leading to increased eIF2α phosphorylation. *B–E*, Western blot analysis and quantitation of phospho-eIF2α, total eIF2α, ATF4, phospho-PERK, and total PERK in NRVMs treated with OSMI-1 (25 μM) with or without the GCN2 inhibitor GCN2iB (10 μM) for 6 h. *F–I*, Western blot analysis and quantitation of phospho-eIF2α, total eIF2α, ATF4, phospho-PERK, and total PERK in NRVMs treated with OSMI-1 (25 μM) with or without the PERK inhibitor GSK2606414 (10 μM) for 6 h. *J–L*, Western blot analysis and quantitation of phospho-eIF2α, total eIF2α, phospho-PERK, and total PERK in NRVMs treated with OSMI-1 (25 μM) with or without the PKR inhibitor PKR-IN (10 μM) for 6 h. *M–N*, Western blot analysis and quantitation of O-GlcNAc levels in NRVMs treated with OSMI-1 (25 μM) with or without the PKR inhibitor PKR-IN (10 μM) for 6 h. Comparisons across groups were performed with one-way ANOVA followed by Tukey's *post hoc* test. ns: not significant, ∗*p* < 0.05, ∗∗*p* < 0.01, ∗∗∗*p* < 0.001, ∗∗∗∗*p* < 0.0001. Complete ANOVA statistics are reported in Table S4.

To gain a better understanding of the upstream mechanisms connecting OGT inhibition and eIF2α phosphorylation, we used available EIF2AK inhibitors to identify the eIF2α kinase responding to OGT inhibition (Fig. 4*A*). Using the small-molecule inhibitor for EIF2AK4/GCN2, GCN2iB (72), we observed a significant reduction in eIF2α phosphorylation both at baseline and in OGT-inhibited conditions (Fig. 4, *B* and *C*). The OSMI-1-induced increase in Atf4 was significantly decreased in OSMI-1/GCN2iB co-treated cells (Fig. 4, *B* and *D*). Furthermore, we observed that OSMI-1 alone was moderately but significantly inducing the relative phosphorylation of EIF2AK3/PERK (Fig. 4, *B* and *E*, compare the blue with the green bar). Interestingly, GCN2iB was potent at inhibiting PERK phosphorylation both at baseline and in OSMI-1-treated conditions (Fig. 4, *B* and *E*). Next, we examined the effect of PERK inhibitor GSK2606414. As shown in Figure 4, *F* and *G*, GSK2606141 did not alter the phosphorylation of eIF2α either at baseline or in OSMI-1-treated conditions and similarly, the upregulation of Atf4 was not impacted significantly by PERK inhibition (Fig. 4, *F* and *H*). As expected, however, GSK2606414 strongly suppressed the phosphorylation of PERK both at baseline and in OSMI-1-treated conditions (Fig. 4, *F* and *I*). These findings suggest that although PERK phosphorylation is upregulated in OSMI-1-treated cells, it is not the key kinase that participates in eIF2α phosphorylation and downstream ISR activation. Next, we also examined the impact of EIF2AK2/PKR inhibition on eIF2α phosphorylation. As shown in Figure 4, *J* and *K*, the compound PKR-IN-C16 was ineffective in reducing eIF2α phosphorylation either at basal or OSMI-1-treated conditions. In fact, we observed a strong induction of eIF2α phosphorylation by PKR-IN-C16 alone (Fig. 4, *F* and *I*, compare blue and red bars). This finding is consistent with a recent report demonstrating that PKR-IN-C16 can activate GCN2 in certain contexts (73). On the other hand, we found that PKR-IN-C16 did not significantly affect the phosphorylation of PERK either at baseline or in OGT-inhibited conditions (Fig. 4, *J* and *L*). It is noteworthy that in these experiments, the O-GlcNAc levels were significantly decreased by OSMI-1, but the PKR-IN-C16 did not have any effect on O-GlcNAc (Fig. 4, *M* and *N*).

Taken together, these findings indicate that GCN2 is likely the kinase responsible for the upregulation of eIF2α phosphorylation in O-GlcNAc depleted conditions. While we did observe activation of PERK, the contribution of this kinase to the observed eIF2α phosphorylation is not substantial. It is noteworthy that we observed an inhibitory effect of GCN2iB on PERK phosphorylation that could be an off-target effect of GCN2iB. Furthermore, it should be noted that even GCN2iB can, at low concentrations, activate instead of inhibit GCN2 (74).

### siRNA-mediated knockdown of EIF2AKs supports the central role of GCN2 in eIF2α phosphorylation

**Figure 5:**
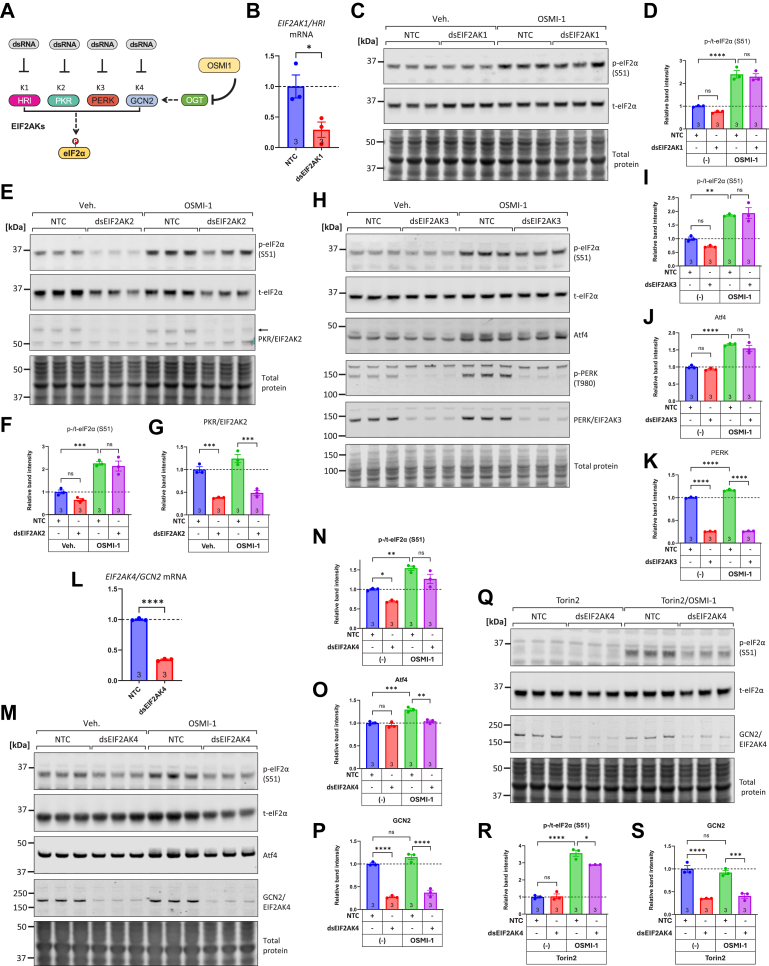
**Knocking-down upstream eIF2α kinases with siRNA supports that EIF2AK4/GCN2 is a key kinase responsible for eIF2α phosphorylation, during OGT inhibition.***A*, schematic depicting the four eIF2α kinases: HRI (EIF2AK1), PKR (EIF2AK2), PERK (EIF2AK3), and GCN2 (EIF2AK4), their respective dsRNAs (Dicer substrate small interfering RNAs) used for knockdown, and the potential impact of OGT inhibition by OSMI-1 in activating one or more of these kinases leading to increased eIF2α phosphorylation. *B*, NRVMs were transfected with 20 nM non-targeting control (NTC) dsRNA, or dsRNA targeting rat HRI/EIF2AK1. 48 h later cells were processed for RNA isolation and mRNA quantification with qPCR. *C–D*, NTC and EIF2AK1 dsRNAs were transfected to NRVMs and 48 h later the cells were treated with or without OSMI-1 (25 μM) for 6 h. Samples were processed for Western blot to assess the levels of phospho- and total eIF2α. *E–G*, NRVMs were transfected with 20 nM NTC or dsRNA targeting rat PKR/EIF2AK2. 48 h after transfection/knockdown, the cells were incubated with or without OSMI-1 (25 μM) for 6 h, and samples were collected for Western blot to assess the levels of phospho-eIF2α, total eIF2α, and PKR. *H–K*, NRVMs were transfected with 20 nM NTC or dsRNA targeting rat PERK/EIF2AK3. After 48 h of transfection/knockdown, the cells were incubated with or without OSMI-1 (25 μM) for 6 h, and samples were collected for Western blot to assess the levels of phospho-eIF2α, total eIF2α, ATF4, phospho-PERK, and total PERK. *L*, NRVMs were transfected with NTC, or EIF2AK4/GCN2-targeting dsRNA. 48 h later cells were processed for RNA isolation and quantification of *EIF2AK4/GCN2* mRNA with qPCR. *M-P*, NRVMs were transfected with 20 nM NTC or dsRNA targeting rat GCN2/EIF2AK4. After 48 h of transfection/knockdown, the cells were incubated with or without OSMI-1 (25 μM) for 6 h, and samples were collected for Western blot to assess the levels of phospho-eIF2α, total eIF2α, ATF4, phospho-GCN2, and total GCN2. *Q–S*, same as in *M*–*P*, but after 48 h of siRNA transfection the cells were exposed to Torin2 (1 μM) with or without OSMI-1 (25 μM) for 6 h, after which cells were harvested and protein extracted for western blots. Comparisons in *B* and *L* used unpaired Student’s t-tests. In all other *panels*, comparisons across groups were performed using two-way ANOVA with Tukey's *post hoc* test. ns: not significant, ∗*p* < 0.05, ∗∗*p* < 0.01, ∗∗∗*p* < 0.001, ∗∗∗∗*p* < 0.0001. Complete ANOVA statistics are reported in Table S5.

Next, we pursued the genetic knockdown of the kinases either individually or in combinations to assess their contributions to eIF2α phosphorylation in OGT-inhibited conditions (Fig. 5*A*). We first knocked down EIF2AK1/HRI, for which no small molecule inhibitors are available. As shown in Figure 5*B*, transfection with HRI-targeting dsRNA significantly reduced the HRI mRNA levels to ∼29% compared to NTC-treated control cells. However, HRI knockdown did not affect the phosphorylation of eIF2α either at baseline or in OGT-inhibited conditions (Fig. 5, *C* and *D*). To demonstrate that knockdown of HRI was important in eIF2α phosphorylation, we used the HRI-activator sodium arsenite (NaAsO_2_, Fig. S3*A*). As shown in Fig. S3, *B* and *C*, sodium arsenite robustly induced the phosphorylation of eIF2α and importantly, transfection with HRI-targeting dsRNA significantly decreased that response. Given the strong induction in eIF2α phosphorylation caused by sodium arsenite, we examined whether other EIF2AKs were activated. A combined knockdown experiment of both EIF2AK2/EIF2AK3 (PKR/PERK) showed that although a 60% and 70% reduction was achieved for PKR and PERK kinases respectively, this did not impact the degree of eIF2α phosphorylation caused by sodium arsenite (Fig. S3, *D*–*G*). Lastly, the protein levels of GCN2 were not affected by these manipulations (Fig. S3, *D* and *H*).

Having demonstrated that HRI is not a major contributor to eIF2α phosphorylation in OGT-inhibited conditions, we pursued the knockdown of EIF2AK2/PKR. This analysis showed the knockdown successfully depleted the PKR protein levels by more than 50%, however, however this did not significantly affect the relative phosphorylation of eIF2α (Fig. 5, *E*–*G*). To demonstrate that knockdown of PKR was important in eIF2α phosphorylation, we performed a control experiment where PKR activation was induced with the viral RNA-mimetic polyinosinic: polycytidylic acid (poly-I:C, Fig. S3*A*). Treating NRVMs with poly-I:C robustly increased the phosphorylation of eIF2α, and this was significantly decreased in cells transfected with dsRNA, causing a greater than 60 to 70% reduction of PKR (Fig. S3, *I*–*L*).

Next, we pursued the knockdown of EIF2AK3/PERK using its cognate dsRNA. As shown in Figure 5, *H*–*J*, eIF2α phosphorylation (and the accumulation of its downstream target Atf4), were significantly elevated by OSMI-1; however, this response was not changed by PERK knockdown, despite a greater than 60% reduction of PERK protein levels (Fig. 5, *H* and *K*). Taken together, the evidence from these knockdown experiments shows that the EIF2AK depletion approach is functionally successful as it can reduce eIF2α phosphorylation downstream of NaAsO_2_ and poly-I:C; however, since the knockdowns do not reduce eIF2α phosphorylation induced by OSMI-1, we can conclude that EIF2AK1-3 (HRI, PKR, PERK) are not essential mediators of eIF2α phosphorylation in OGT-inhibited conditions.

Finally, we performed the knockdown of EIF2AK4/GCN2 in vehicle- and OSMI-1-treated NRVMs. As shown in Figure 5*L*, transfection with GCN2-targeting dsRNA significantly reduced GCN2 mRNA levels to ∼34% compared to NTC-transfected control cells. Transfection with GCN2-targeting dsRNA caused a significant reduction in baseline phosphorylation levels of eIF2α and a trend, albeit not significant, reduction in the relative levels of phospho-eIF2α, after OSMI-1 treatment (Fig. 5, *M*–*P*). Previously, we showed that mTOR participates in eIF2α phosphorylation downstream of OGT inhibition (Fig. 2) which could be masking the effects of GCN2 knockdown on reducing eIF2α phosphorylation. We therefore performed the GCN2 knockdown experiment in the presence of Torin2 to remove any parallel input from mTOR signaling. Indeed, this experiment revealed that GCN2 knockdown was efficient at significantly decreasing eIF2α phosphorylation in OGT-inhibited conditions (Fig. 5, *Q*–*S*).

### GCN2 activation in OGT-inhibited cells is not due to widespread amino acid starvation

Next, we employed halofuginone, which inhibits glutamyl-prolyl tRNA synthetase (EPRS), causing the buildup of uncharged tRNAs that activate GCN2 (75). Additionally, this inhibition causes the accumulation of free amino acids that in turn activate mTORC1 (76) (Fig. S4*A*). Consistently, we found that halofuginone activates mTORC1 (Fig. S4, *B*–*D*) and GCN2 (Fig. S4, *E*–*H*) in NRVMs, closely mimicking the responses observed after OGT inhibition. Moreover, dual inhibition of both mTOR and GCN2 is necessary to significantly counter the halofuginone-induced eIF2α phosphorylation (Fig. S4, *J*–*M*).

The hexosamine biosynthetic pathway (HBP) is linked with other nutrient metabolizing pathways, including glycolysis, glutamine metabolism, and the tricarboxylic cycle (Fig. S5*A*). Furthermore, it has been shown that the GCN2/eIF2α/ATF4 signaling axis can be activated in conditions of glucose or amino acid starvation to increase the expression of *Gfpt1*, encoding *Gfat1*, the rate-limiting enzyme of HBP (77). Our qPCR gene expression analysis, however, showed that OGT-inhibited NRVMs had a strong reduction in *Gfpt1* mRNA levels, while the expression of other HBP genes (*Gnpnat1*, *Pgm3*, and *Uap1*) remained unchanged (Fig. S5*B*). The expression of *Gls1*, encoding the enzyme that participates in the breakdown of glutamine, and *Gdh*, encoding the enzyme that catalyzes the interconversion of α-ketoglutarate to glutamate, were significantly increased, suggesting a shift in amino acid metabolism (Fig. S5, *A* and *B*). Quantitative targeted metabolomics of amino acid levels in OGT-inhibited NRVMs found significant increases in aspartate and significant decreases in glutamine (Fig. S5*C*). Previous studies showed that restricting glutamine activates the GCN2/eIF2α/ATF4 pathway in cancer cells (78, 79, 80). Therefore, we asked whether supplementation with glutamine could impact the phosphorylation of eIF2α in OGT-inhibited cells. Interestingly, there was no change in the levels of phospho-eIF2α in NRVMs treated with or without additional glutamine (Fig. S5, *D* and *E*). Furthermore, glutamine supplementation did not cause significant increases in O-GlcNAc levels either in basal or OGT-inhibited conditions (Fig. S5, *F* and *G*). Taken together, these findings show that despite activation of the GCN2/eIF2α/ATF4 signaling axis, the levels of *Gfpt1* are decreased. Furthermore, while we observe a reduction in glutamine levels in OSMI-1-treated cells, this does not appear to be a major driving factor causing the observed activation of the GCN2/eIF2α/ATF4 signaling axis.

Finally, we examined the role of eIF2α phosphatases. The catalytic subunit responsible for dephosphorylation of eIF2α is protein phosphatase 1 (PP1) which is targeted to eIF2α *via* protein scaffolds Gadd34 (Ppp1r15a) and CReP (Ppp1r15b) (81) (Fig. S6*A*). First, we targeted the enzymatic activity of PP1 using calyculin A, which caused the expected increase in eIF2α phosphorylation (Fig. S6, *B* and *C*). Next, we used siRNA-mediated targeting of the two scaffold proteins to examine whether this could affect the dynamics of eIF2α phosphorylation in OGT-inhibited conditions. This analysis showed that the constitutive scaffold protein CReP, and not the inducible Gadd34, is likely to be a key participant for baseline eIF2α dephosphorylation (Fig. S6, *D*–*I*). However, depleting CReP causes an increase in eIF2α phosphorylation that is additive to that of OSMI-1 (Fig. S6, *E* and *F*). Taken together these data suggest that while PP1/CReP-mediated dephosphorylation of eIF2α is operational in NRVMs, direct inhibition of that pathway is unlikely to be causing the observed increase in eIF2α phosphorylation during OGT inhibition.

### Genetic knockout of OGT in cardiac myocytes causes progressive cardiac dysfunction and failure that is accompanied by increases in GCN2 and eIF2α protein levels

**Figure 6:**
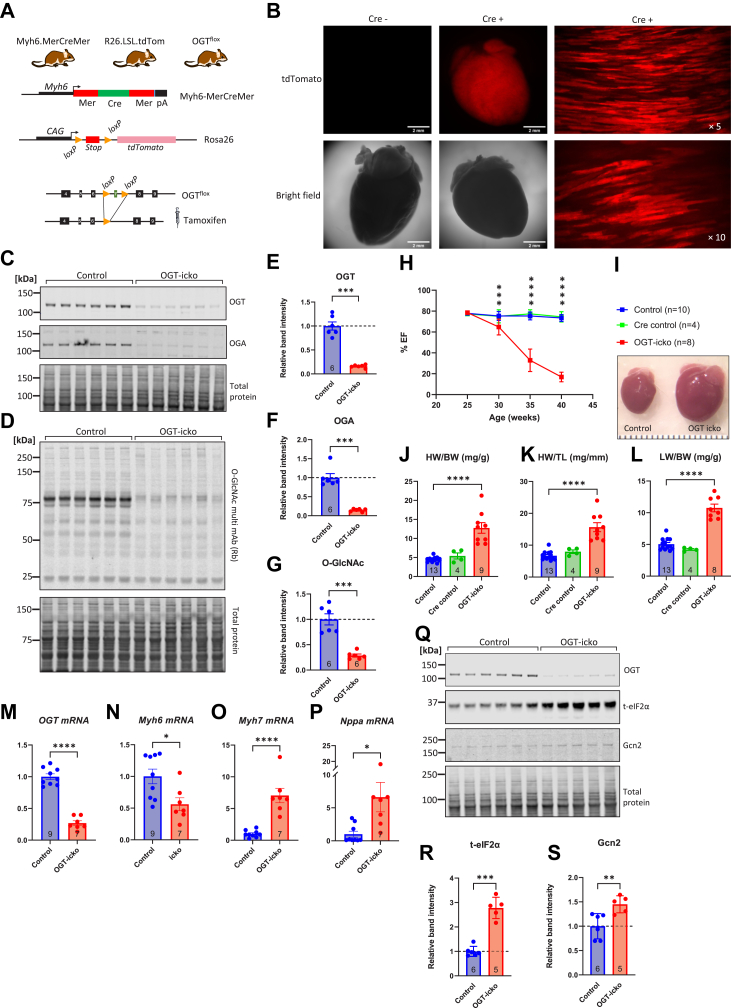
**Genetic ablation of OGT in cardiac myocytes in adulthood causes progressive heart dysfunction and failure that is associated with increased expression of eIF2α and GCN2 proteins.***A*, schematic representation of the genetic model for the selective knockout of O-GlcNAc transferase (OGT) in cardiac myocytes using an inducible Cre/loxP system. Cre recombinase is driven by the Myh6 promoter for cardiomyocyte-specific expression. Cre activity is tamoxifen-inducible due to the presence of two Mer domains (modified estrogen receptor) that promote nuclear localization of Cre upon binding to tamoxifen. Additionally, a tdTomato marker, inserted into the Rosa26 locus, was introduced by cross-breeding. The expression of tdTomato is driven by the ubiquitous CAG promoter (a hybrid promoter derived from the chicken β-actin promoter and the cytomegalovirus enhancer) but is preceded by a lox-stop-lox cassette that is removed only in the presence of Cre in cardiac myocytes. *B*, bright field and fluorescent confocal images from adult hearts negative or positive for Cre that were intraperitoneally injected with tamoxifen (20 mg/kg/day) for 4 days. The images show strong expression of tdTomato protein in cardiac myocytes of Cre-positive mice. Images were captured using a Nikon spinning disk confocal microscope (Nikon Ti-E) equipped with 5× and 10× objectives and a high-speed camera. tdTomato was excited with the 561 nm laser. Scale bars are 2 mm. *C-G*, Mice (5 male and one female per group, aged 12–13 weeks old) were injected with four daily intraperitoneal injections of tamoxifen (20 mg/kg/day), and hearts were collected 15 weeks later. Heart homogenates (20 μg/lane) were analyzed by Western blot for the expression of OGT, OGA, and overall O-GlcNAc levels. *H*, serial echocardiograms were obtained from flox-only control, Cre-only control mice, and OGT inducible cardiomyocyte specific knockout mice (OGT-icko) over the period of 15 weeks after tamoxifen injection. EF: Ejection Fraction, square symbols represent means ± standard deviation. *I*, gross morphological appearance of Control and OGT-icko hearts after 15 weeks of follow-up. *J–L*, heart and lung analysis in Control and OGT-icko hearts. Heart weight (HW), body weight (BW), tibia length (TL), and lung weight (LW) are measured. *M-P*, quantitative real-time PCR analysis of RNA extracted from Control and OGT icko hearts (15 weeks after tamoxifen injection) to assess OGT expression levels and markers of detrimental cardiac remodeling. Myh6: myosin heavy chain 6; Myh7: myosin heavy chain 7; Nppa: natriuretic peptide A. *Q–S*, heart homogenates (20 μg/lane) from Control and OGT-icko mice were analyzed by Western blot for the expression of O-GlcNAc transferase (OGT), eukaryotic initiation factor 2 alpha (eIF2α), and general control nonderepressible 2 (GCN2). Statistical Analysis: Bars represent means ± standard error. Comparisons between two groups were performed using an unpaired two-tailed Student’s *t* test. Comparisons across three groups (*Panels J*–*L*) were conducted using one-way ANOVA with Tukey's *post hoc* test. For the serial echocardiography analysis (*panel H*), a two-way ANOVA was used, followed by Tukey’s correction for multiple comparisons. ∗*p* < 0.05, ∗∗*p* < 0.01, ∗∗∗*p* < 0.001, ∗∗∗∗*p* < 0.0001. Complete ANOVA statistics are reported in Tables S4 and S5.

To extrapolate our findings with NRVMs to the setting of the intact heart, we developed mice with cardiomyocyte-specific targeting of OGT. Here, the OGT gene was inactivated in the adult heart by intraperitoneal tamoxifen injections. The genetic design involves the crossing of mice with conditional OGT alleles (OGT^flox^) with mice harboring the MerCreMer transgene that responds to tamoxifen and is driven by the cardiomyocyte-specific promoter *Myh6* (Fig. 6*A*). A third transgene encoding for a reporter cassette (inducible tdTomato) knocked into the Rosa26 locus was also introduced by cross-breeding (Fig. 6*A*). This approach allowed the direct visualization of recombinant cardiomyocytes by observing the expression of tdTomato in the myocardium of the triple transgenic hearts, injected with tamoxifen (Fig. 6*B*). Examination of control vs. OGT-icko hearts 15 weeks after tamoxifen injection revealed a robust reduction in OGT protein levels in icko hearts (84% lower than control, Fig. 6, *C*–*E*). Consistent with reports indicating a downregulation of OGA when OGT is inhibited (82, 83), we also observed a significant reduction in OGA protein levels (85% lower than control, Fig. 6, *C* and *F*). Nevertheless, despite the concerted downregulation of both OGT and OGA proteins, there was a significant reduction in overall O-GlcNAcylation in whole heart extracts (70% lower than control, Fig. 6, *D* and *G*). These changes were accompanied by a significant impact on overall cardiac function, as the OGT-icko hearts exhibited a dramatic deterioration of heart function over the course of 15 weeks (Fig. 6*H*). A reduction in ejection fraction (EF) in OGT-icko mice was observed by 5 weeks after tamoxifen injection, consistent with previous reports (84, 85) and further follow-up revealed a detrimental cardiac remodeling with evident cardiac dilation at 15 weeks after tamoxifen injection (15w OGT-icko, Fig. 6*I*). Indeed, the OGT-icko hearts exhibited a significant increase in mass (both normalized to body weight or tibia length, Fig. 6, *J* and *K*), and this was accompanied by a significant increase in lung edema (increased lung weight/body weight, Fig. 6*L*), an indicator of heart failure.

Further molecular analysis of the 15w OGT-icko hearts revealed the expected reduction in OGT mRNA (86% lower than control, Fig. 6*M*), and we could also observe the myosin isoform switch that occurs in failing hearts, with reduction of isoform Myh6 and upregulation of Myh7 (Fig. 6, *N* and *O*). Finally, we found that the levels of Nppa, another biomarker of detrimental cardiac remodeling, were significantly increased (Fig. 6*P*) in 15w OGT-icko hearts. We also examined markers of the ISR by Western blot in 15w OGT-icko hearts, where we observed a significant increase in eIF2α protein levels (2.8-fold higher than control, Fig. 6, *Q* and *R*) and, similarly, an increased expression in GCN2 (45% higher than control, Fig. 6, *Q* and *S*). In summary, the genetic approach employed here efficiently depletes OGT and O-GlcNAcylation in the adult heart, that is characterized by the upregulation of molecular markers of heart failure but also expression levels of eIF2α and GCN2. The upregulation of the latter is consistent with the aberrant activation of the ISR when OGT and O-GlcNAcylation are suppressed in cardiac myocytes.

### Treatment with the ISR inhibitor ISRIB prevents detrimental cardiac remodeling caused by cardiomyocyte OGT deficiency

**Figure 7:**
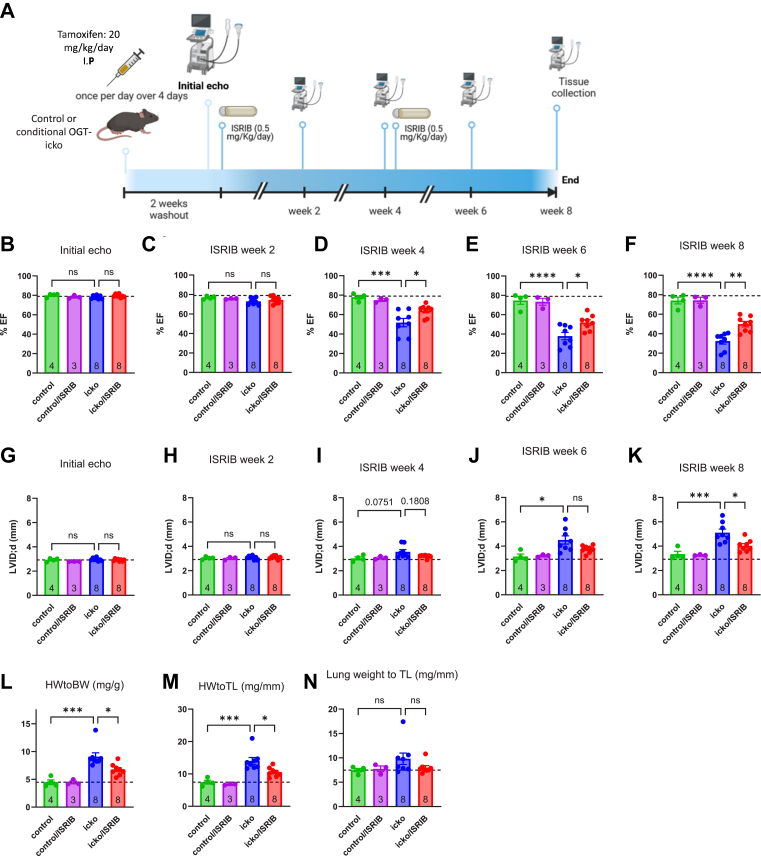
**Treatment with the ISR inhibitor ISRIB prevents the development of dilated cardiomyopathy in OGT-icko mice.***A*, timeline showing tamoxifen injection to produce Control and OGT-icko. The mice (male, aged 18 weeks old) were analyzed by echocardiography 2 weeks later and randomized to undergo implantation of osmotic pumps delivering ISRIB (0.5 mg/kg/day). Following the first 4-week period the ISRIB treatment continued for another 4 weeks with a second round of pump implantations. Serial echocardiograms were obtained before the first pump implantation and then every 2 weeks. *B–F*, each *panel* shows the results of ejection fraction (EF) at the beginning and then at 2, 4, 6, and 8 weeks of ISRIB infusion. *G–K*, Each *panel* shows the results of *left* ventricular internal diameter during diastole (LVID;d) at baseline, 2, 4, 6, and 8 weeks of ISRIB treatment. Four groups were used: Control with or without ISRIB and OGT-icko with or without ISRIB. The dashed line indicates the mean value for EF and LVID;d in control mice at the initial echo. *L–N*, Morphometric parameters of hearts from the four different groups at the end of the 8-weeks ISRIB experiment. Statistical Analysis: Bars represent means ± standard error. Comparisons across groups were performed using two-way ANOVA with Tukey's *post hoc* test. The number of mice per group is indicated in the bars ns: not significant, ∗*p* < 0.05, ∗∗*p* < 0.01, ∗∗∗*p* < 0.001, ∗∗∗∗*p* < 0.0001. Complete ANOVA statistics are reported in Table S5.

Given our findings with OGT inhibition in NRVMs and the detection of elevated levels of ISR members GCN2 and eIF2α, we asked whether suppressing the ISR could be beneficial for the OGT-deficient hearts. To address this question, control or conditional OGT knockout mice received tamoxifen injections and 2 weeks later were randomized to receive treatment with or without ISRIB infusion (0.5 mg/kg/day). Serial echocardiography was performed to assess changes in cardiac function over a period of 8 weeks of ISRIB treatment (Fig. 7*A*). The EF of the OGT-icko mice was not significantly different from control mice at the onset of ISRIB infusion (2 weeks after tamoxifen injection, Fig. 7*B*) and at 2 weeks of ISRIB infusion (Fig. 7*C*). However, at 4 weeks of ISRIB infusion, OGT-icko hearts exhibited decreased EF, whereas OGT-icko/ISRIB hearts did not show a significant decrease in EF (Fig. 7*D*). By 6 weeks of ISRIB infusion, the OGT-icko/ISRIB group showed significantly improved function compared to OGT-icko hearts (Fig. 7*E*), and these differences became even more significant by the end of the study at 8 weeks of ISRIB (Fig. 7*F*). Monitoring for chamber dilation using the diastolic diameter LVID;d, revealed similar trends (Fig. 7, *G*–*J*) and after 8 weeks of ISRIB infusion there was a significant difference in LVID;d, between untreated and ISRIB-treated OGT-icko hearts (Fig. 7*K*).

As expected, the OGT-icko hearts exhibited significant increases in cardiac mass compared to control hearts (normalized to body weight, or tibia length, Fig. 7, *L* and *M*); however, the OGT-icko/ISRIB hearts had significantly lower ratios (Fig. 7, *L* and *M*), consistent with less adverse cardiac remodeling. In this 10w post-tamoxifen study, lung weights were not significantly increased in OGT-icko hearts (Fig. 7*N*), likely because the duration of the overall protocol was shorter by 5 weeks compared to the 15-week follow-up study (Fig. 6). Collectively, these experiments show that infusion with ISRIB effectively delays the adverse cardiac remodeling that occurs with OGT deficiency, implicating the ISR as a potentially maladaptive mechanism in this long-term experimental setting.

### ISRIB-mediated protection against OGT-cardiomyopathy is associated with reduced PERK and eIF2α protein levels and further increases in mTOR

**Figure 8:**
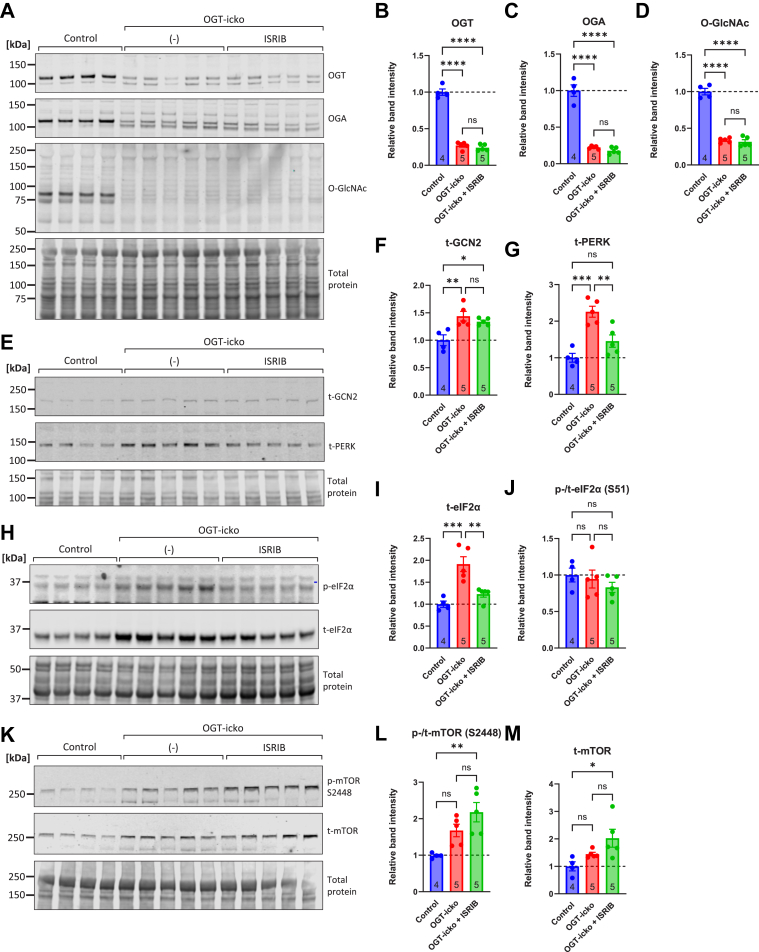
**ISRIB-mediated protection against OGT-cardiomyopathy associates with reduction in PERK and eIF2α protein levels and sustained increases in GCN2 and mTOR.***A–D*, Homogenates (20 μg/lane) were analyzed by Western blot for the expression of OGT, OGA, and overall O-GlcNAc levels. *E–G*, hearts were analyzed by Western blot for the expression of EIF2AKs PERK and GCN2. *H–J*, Western blot analysis of phospho- and total eIF2α, either as ratio or total eIF2α levels alone. *K-M*, Western blot analysis of phospho- and total mTOR in the same heart homogenates. Comparisons across three groups were performed using one-way ANOVA with Tukey's *post hoc* test. The number of mice per group is indicated in the bars ns: not significant, ∗*p* < 0.05, ∗∗*p* < 0.01, ∗∗∗*p* < 0.001, ∗∗∗∗*p* < 0.0001. Complete ANOVA statistics are reported in Table S4.

Given the significant impact of ISRIB against the development of OGT-cardiomyopathy (Fig. 7), we sought to investigate the molecular changes that underlie this effect. OGT, OGA and O-GlcNAc levels were all reduced in the OGT-icko hearts, and this was not affected by the treatment with ISRIB (Fig. 8, *A*–*D*). Next, we investigated members of the ISR. As previously shown, GCN2 levels rose by 40 to 50% in OGT-icko hearts; however, the treatment with ISRIB did not significantly change that (Fig. 8, *E* and *F*). Interestingly, the levels of PERK were more than two-fold elevated in OGT-icko hearts and this was prevented by treatment with ISRIB (Fig. 8, *E* and *G*). Similarly, total eIF2α levels were significantly increased in OGT-icko hearts, and ISRIB treatment prevented that rise (Fig. 8, *H* and *I*). The levels of phospho-eIF2α followed the same pattern (Fig. 8*H*); however, after normalizing the phospho-over the total-eIF2α, the changes were not significant between the groups (Fig. 8, *H* and *J*). These data indicate that in this setting, the regulation in eIF2α signaling is mainly driven by total protein abundance, rather than its relative phosphorylation levels. Finally, we also examined the expression of mTOR, which showed that both phospho- and total mTOR tended to increase in OGT-icko hearts and ISRIB treatment further potentiated these changes (Fig. 8, *K*–*M*).

## Discussion

**Figure 9:**
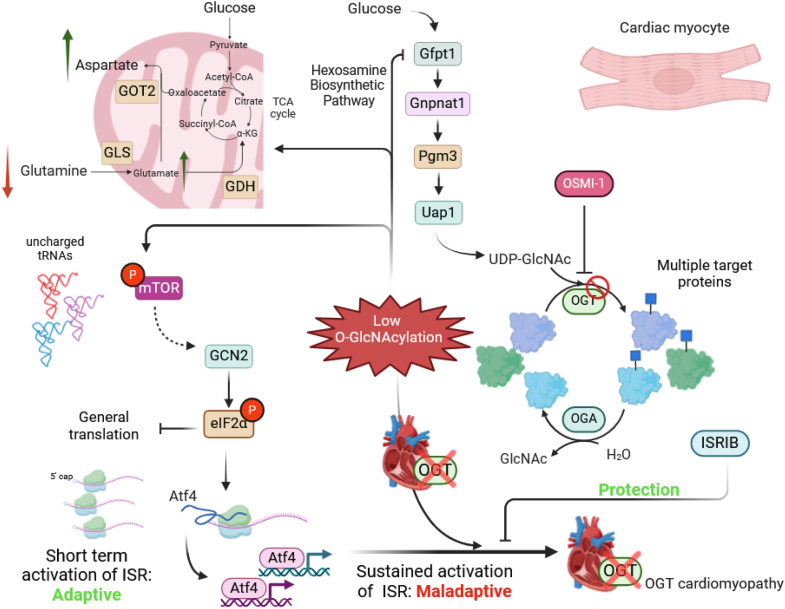
**Working model summarizing the findings of the study and highlighting the relationship between O-GlcNAcylation and ISR activation.** Lowering O-GlcNAcylation triggers the ISR *via* the GCN2/eIF2α signaling axis. This causes a reduction in general translation but accumulation of transcription factor Atf4. Activation of GCN2 by low O-GlcNAcylation requires mTOR. Amino acid levels are not substantially decreased in OGT-inhibited cardiomyocytes, except for glutamine. It is thus unlikely that a surge in uncharged tRNAs is activating GCN2 in this context. Concurrently with glutamine reduction, glutamate and aspartate levels increase, highlighting a potential metabolic switching in mitochondria. Lowering O-GlcNAcylation causes the downregulation of *Gfpt1* mRNA which encodes the rate-limiting enzyme of the HBP *Gfat1*. Hearts with cardiomyocyte-specific knockout of OGT exhibit severe dysfunction (cardiomyopathy) over the course of 15 weeks of knockout that is significantly delayed by ISRIB, highlighting the importance of the ISR in the development of OGT cardiomyopathy.

O-GlcNAcylation is known to regulate a multitude of intracellular signaling pathways, including those closely related to stress sensing and stress adaptation (7, 11). Previously, we demonstrated that O-GlcNAcylation impacted the main effectors of MAPK signaling, a key pathway involved in cardiomyocyte response and adaptation to stress (33). In this work, we demonstrate that O-GlcNAcylation also impacts the integrated stress response (ISR). Our findings indicate that depleting cells of O-GlcNAcylation triggers a signaling axis that involves activation of the nutrient-sensitive kinase GCN2, which in turn phosphorylates eIF2α, leading to inhibition of cap-dependent mRNA translation and facilitating the translation of stress-adaptive mRNAs, such as Atf4 (Fig. 9). While the upstream events that lead to the activation of the GCN2/eIF2α/Atf4 signaling axis remain to be fully resolved, we have found that the nutrient sensor, mTOR, activates this signaling axis when O-GlcNAcylation levels are low (Fig. 9). As loss of OGT leads to HF, we examined the efficacy of ISRIB in preventing cardiomyopathy in OGT-deficient mice. Indeed, the long-term infusion of ISRIB demonstrated a significant effect in delaying the progression of heart failure in OGT-deficient mice (Fig. 9), linking O-GlcNAcylation and the ISR in an *in vivo* model of cardiac pathophysiology.

The importance of OGT/O-GlcNAc signaling system has become increasingly recognized in the regulation of protein synthesis at multiple levels, including direct modification on ribosome subunits (86, 87), translation initiation factors (88, 89), and nascent polypeptides as they are being synthesized (90). Furthermore, OGT appears to associate with ribosomes (86), and translation factors (91). Thus, it is reasonable to expect that depleting cells of O-GlcNAcylation would impact protein synthesis. While our investigation focused on the mechanisms leading to eIF2α phosphorylation and the switch in translation from cap-dependent to cap-independent, it should be noted that preventing that switch with ISRIB did not overcome the translational blockade that is caused by OGT inhibition, both at baseline and in PE-stimulated conditions. It is therefore clear that other mechanisms beyond the phosphorylation of eIF2α are operating in O-GlcNAc-depleted cells that promote translational inhibition. One likely possibility is that the concerted depletion from O-GlcNAcylation in ribosomal subunits, translation factors, or nascent polypeptides creates the observed effect. Along these lines, gene ontology analysis on data from published O-GlcNAcomes indicates that proteins participating in the translational machinery, including ribosomal subunits, and factors regulating translational initiation, elongation, and termination are commonly enriched (92).

Apart from the translational machinery, the ubiquitin proteasome system (UPS), represents another target of O-GlcNAcylation (63, 93). Critically, the lack of OGT in embryonic stem cells resulted in overactive UPS leading to excess protein recycling and elevated amino acid levels, which in turn caused the activation of mTOR (68). This mechanism presents both similarities and differences with our findings. For example, treating cells with the proteasome inhibitor MG132 did not reverse the impact of OSMI-1 on protein synthesis, indicating that the proteasome activity is not critically implicated in this context. Furthermore, the amino acid levels in OGT-inhibited cells were not uniformly increased as it was observed with the OGT-KO stem cells (68). Therefore, it is unlikely that the activation of mTOR that we observe is due to overactive proteasome and excess amino acids. It is noteworthy that most of the published evidence suggests a positive, rather than a negative regulation of mTOR by OGT. For example, investigations in obese tissues and cancer cell lines show a positive correlation between O-GlcNAcylation and mTOR phosphorylation (94), while OGT inhibition in cortical neurons increases autophagy markers *via* mTOR inhibition (95). Furthermore, there is support for an indirect regulation of mTOR by OGT, *via* AMPK and TSC1/2. In this mechanism, OGT causes the O-GlcNAcylation of AMPK, which in turn is blocked from phosphorylating TSC1/2, ultimately causing the de-repression of mTORC1 (96). Lastly, a recent study showed that OGT positively regulates mTOR signaling by O-GlcNAcylating the mTORC1 subunit Raptor, promoting pathway activation (69). Taken together, these data suggest that OGT is generally a positive regulator of mTOR signaling. Therefore, the precise mechanism leading to mTOR activation in OGT-inhibited cardiac myocytes described here remains to be determined.

GCN2 is activated by uncharged tRNAs, accumulating during amino acid depletion (97, 98). Previous reports show that lack of O-GlcNAcylation can affect the function of amino acid-tRNA synthetase (99), which could lead to elevated uncharged tRNAs and in turn activation of GCN2. While this is a possible scenario, the total levels of amino acids do not appear to change uniformly in OSMI-1-treated cells, leaving room for alternative possibilities. GCN2 has a ribosome-interacting domain, and it is found to co-migrate with ribosomes on sucrose gradients in yeast (100, 101). Importantly, emerging evidence indicates that GCN2 can be activated by mechanisms that do not require uncharged tRNAs as an activating ligand, but rather involve the ribosome. These include induction of GCN2 by stalled ribosomes (102, 103), activation through interactions with the ribosomal P-stalk (104, 105), or due to ribosomal collisions (106). We did not observe widespread changes in amino acid levels in OSMI-1-treated cardiomyocytes (Fig. S4), suggesting that the pool of uncharged tRNAs in these cells is unlikely to be substantially altered. Thus, it is possible that the activation of GCN2 that we observed here is due to abnormalities in ribosomal function leading to colliding or stalled ribosomes. Along these lines, it is noted that OGT-deficient MEFs show defective polysome formation in sucrose gradients (89), indicating potential defects in the processes of initiation, elongation and termination. Ultimately, future work will be needed to determine whether lack of O-GlcNAcylation has any impact on ribosomal stalling, colliding, or the signaling function of the ribosomal P-stalk, and whether these effects can activate GCN2 and the downstream signaling axis.

The finding that Torin2 inhibits OSMI-1-induced eIF2α phosphorylation, implicates mTOR upstream of GCN2 in our working model (Fig. 9). The interplay between GCN2 and mTOR is complex, since the two kinases are at the cross-roads of regulating processes responsive to scarcity, or abundance of nutrients, respectively. Most studies indicate that GCN2 negatively impacts mTOR signaling, which can be advantageous during times of nutrient scarcity when it’s essential to suppress the anabolic effects of mTOR (107). For example, during prolonged nutrient starvation, the GCN2/eIF2α/Atf4 signaling pathway enhances the expression of Sestrin2 and Redd1, both of which directly inhibit mTORC1 (108, 109). Additionally, Atf4 can transcriptionally upregulate LARP1 (110), an mRNA-binding protein that counteracts the effects of mTORC1 on selective mRNA translation (111). Finally, GCN2 is found to phosphorylate FBXO22, which causes the ubiquitination and inhibition of mTORC1 (112). While these results show that GCN2 operates upstream of mTORC1, there is some other emerging evidence showing that mTORC1 can exert regulation onto GCN2 by direct phosphorylation (113). In this context, acute amino acid depletion in cells deficient in mTOR suppressors TSC2 and NPRL2 causes overactivation of mTOR, which in turn phosphorylates GCN2 to activate the GCN2/eIF2α/Atf4 signaling axis (113). These findings appear similar to those observed here with OGT-inhibited cardiomyocytes, although key differences exist, for example, in the OGT-inhibited cardiomyocytes there is no deficiency of mTOR suppressors, and furthermore, there is no apparent amino acid shortage. Therefore, a different mechanism might be operational in our setup that connects mTOR with the GCN2/eIF2α/Atf4 signaling axis. Interestingly, it has been recently reported that mTOR inhibitors can reverse GCN2 activation (114). While these findings are consistent with our observations, the mechanism appears to be different, since it implicates leucine scarcity and the reversal of it by mTOR inhibition. In our setting, we did not observe leucine depletion, and as discussed above, GCN2 activation is unlikely to be due to amino acid starvation.

In addition to its effects on GCN2/eIF2α/Atf4 signaling axis, OGT inhibition appears to have a significant impact on the expression of metabolic genes, as shown by the analysis of transcripts encoding enzymes of the HBP and those involved in glutamine metabolism (Fig. 9). Interestingly, previous reports show that *Gfpt1* is transcriptionally activated by Atf4 (77, 115). In addition to Atf4, Xbp1s is another transcriptional factor known to activate the expression of multiple HBP genes, including *Gfpt1* (116, 117). Our data show increased expression of both Atf4 and Xbp1s transcripts, yet the expression of *Gfpt1* is downregulated. This points to other transcriptional mechanisms, potentially more dominant than Atf4 and Xbp1s, that might be driving the observed downregulation. There is currently a scarcity of mechanisms known to suppress the expression of *Gfat1* or other HBP pathway genes, although the activity of the Gfat1 enzyme is known to be negatively regulated by feedback inhibition by UDP-GlcNAc (118). The downregulation of these genes may indicate an underlying metabolic shift that is activated in OGT-inhibited cells. Such a shift could be consistent with the upregulation of *Gls1* (encodes the enzyme catalyzing the breakdown of glutamine to glutamate), as well as an increased expression of *Gdh*, (encodes the enzyme catalyzing the interconversion of α-ketoglutarate to glutamate). Elucidating the underlying mechanisms and consequences of such a metabolic switch in OGT-inhibited cardiomyocytes merits further investigation.

The inducible cardiomyocyte-specific deletion of OGT in adult cardiomyocytes has been previously pursued and, consistent with our findings, defects in cardiac function do not become evident until about 3 to 4 weeks after knockout, which then become progressively more severe, leading to dilated cardiomyopathy (84). This is consistent with other reports on OGT-icko models that examined a shorter duration of knockout (2–4 weeks) and that did not find significant impairments in baseline function (17, 22). While these studies focused on cardiac stresses such as post-infarction heart failure and pressure overload, the findings in 2-weeks unstressed OGT-icko hearts showed no apparent cardiomyocyte hypertrophy, fibrosis, or cell death, although phospholamban and cardiac troponin I phosphorylation were reduced (17). In another model of OGT-icko (∼3 weeks), it was found that cardiomyocyte Ca^2+^ handling was impaired with reduced expression of voltage-gated calcium channels and increased protein neddylation and ubiquitination (85). In contrast, excessive elevation of O-GlcNAcylation in cardiomyocytes throughout development and early adulthood can be detrimental to the heart, through mechanisms that involve reduced phosphorylation of phospholamban, increased occurrence of calcium sparks and reduced mitochondrial complex I activity (24). Finally, moderate elevation of O-GlcNAcylation by means of dominant negative OGA overexpression in adulthood did not significantly impact heart function at 2 weeks, but caused detrimental cardiac remodeling at 24 weeks characterized by reduced expression of Ca^2+^ handling, mitochondrial, glucose and fatty acid oxidation genes (119). While highly informative, these models did not identify any direct connections with the ISR, such as the increased expression of GCN2, PERK, and eIF2α observed here, so the generalizability of our findings in different models of aberrant cardiac O-GlcNAcylation remains to be examined in future studies.

The role of the ISR has been previously investigated in the basal and stressed heart. For example, activation of the PERK/eIF2α signaling axis confers protection against acute ischemia/reperfusion injury (59). The HRI/eIF2α/Atf4 signaling axis of the ISR was also shown to be activated in the embryonic, or early postnatal heart, as a protective response against mitochondrial dysfunction (120). Consistently, ISR-mediated activation of Atf4 serves a protective role in the context of tafazzin deficiency and mitochondrial dysfunction (121), where Atf4 drives a metabolic switch that upregulates one-carbon metabolism and the biosynthesis of glutathione. Similarly, Atf4 is shown to regulate the expression of NADPH-generating enzymes to affect redox and antioxidant-related pathways and is protective against cardiac pressure overload (122). While these reports indicate a generally protective effect of the ISR in the heart, there is also evidence that excess ISR activation might be detrimental. For example, the cardiotoxic cancer drug ponatinib was found to activate the GCN2/eIF2α/Atf4 signaling axis in hiPSC-CMs and ISRIB was protective against cardiomyocyte death (123). This study also demonstrated that ISRIB was cardioprotective in a cardiotoxicity/comorbidity mouse model (123). ISRIB was also found to be protective in a model of post-infarct atrial fibrillation (57). Our findings show for the first time that ISRIB has protective effects in the context dilated cardiomyopathy due to OGT deficiency.

Interestingly, the OGT inhibitor TMG appears to potentiate the ISR in neuroblastoma cells and in mouse brains (124). In this model, prolonged exposure to TMG combined with mild mitochondrial stress leads to the activation of the HRI/eIF2α/Atf4 signaling axis of the ISR. However, OGT knockdown in the same study was also shown to be a potent inducer of the ISR. Finally, the ISR is activated in organoid or transgenic mouse models of Alzheimer's disease and treatment with TMG failed to further alter the ISR (124). Taken together, it appears that changes in O-GlcNAcylation either above or below a steady state threshold are potent inducers of the ISR in neurons and brains. Collectively, these studies highlight the close relationship between O-GlcNAcylation and ISR, consistent with the findings we present here. It would be therefore interesting to test the efficacy of ISRIB in other conditions that are characterized by aberrant O-GlcNAc signaling, such as hyperglycemia, diabetes (21, 30, 125, 126), and inherited forms of X-linked intellectual disability (127, 128, 129, 130).

In summary, we have uncovered a functional relationship between O-GlcNAcylation and ISR signaling. Specifically, we have shown that the GCN2/eIF2α/Atf4 signaling axis is activated upon O-GlcNAcylation inhibition in cardiomyocytes. The upstream mechanisms that activate GCN2 when O-GlcNAcylation is reduced remain to be further elucidated, although our data point to a mechanism that involves protein translation and the signaling mediator mTOR. Furthermore, we have demonstrated that absence of cardiomyocyte OGT is detrimental to cardiac function and this effect can be ameliorated if animals are treated with the small molecule ISRIB. The work presented here highlights the responsiveness of the ISR to either short-term or long-term reductions of O-GlcNAcylation in cells and intact hearts. We anticipate that studies that define the molecular underpinnings of the ISR and O-GlcNAc will help identify effective approaches to forestall the progression of dilated cardiomyopathy or other types of cardiac dysfunction.

## Experimental procedures

### Sex as biological variable

For the initial characterization of the heart failure phenotype in OGT-deficient hearts, our study examined male and female animals, and similar findings were found for both sexes. For the subsequent characterization of the efficacy of ISRIB to impact heart failure, our study used male mice. We expect our findings to be relevant for both males and females.

### Materials

A detailed list of key reagents including their identifiers, vendors, and catalog numbers is provided in Table S1. Additional information on other reagents can also be found in the description of methods in the text below. The primer sequences used in quantitative real-time PCR are shown in Table S2. Artwork in some figures was created using BioRender. Mouse genotyping was performed using 1 to 2 mm of tail tissue collected from mice at, or before weaning age. The tissue samples were digested overnight at 55 °C in a lysis buffer (DirectPCR, Cat. No. 102-T, Viagen) containing proteinase K (250 μg/ml). Following digestion, the solution was heated at 85 °C for 1 h to inactivate the enzyme. Samples were briefly centrifuged, and the supernatant was diluted 20 × before being used for PCR analysis to determine the genotype. The primer sequences for the genotyping of mice are shown in Table S3.

### Primary neonatal myocyte culture and treatments

Neonatal rat ventricular myocytes (NRVMs) were isolated from P0-P1 rats of either sex using enzymatic dissociation, according to a standard methodology as previously described (131, 132). Cardiomyocytes at 0.5 × 10^6^ cells/ml were seeded on culture plates pre-coated with 0.1% porcine gelatin (Cat. No. G1890, Sigma) and incubated at 37 °C, 5% CO_2_ for 24 h. On the next day, the cells were washed twice with prewarmed PBS, and the medium was switched to DMEM (Cat. No. 10-013-CV, Corning, 4.5 g/L glucose) supplemented with insulin transferrin selenium (ITS, Cat. No. 51500056, Gibco). Following 24 h of incubation in serum-free media, the cells were stimulated with the indicated agonists and/or inhibitors for the specified durations for each experiment (see also main text and/or figure legends). In all experiments where agonists or inhibitors were dissolved in DMSO, corresponding vehicle controls (≤0.1% DMSO) were included. In experiments for gene knockdown, cells were transfected with a lipofectamine/siRNA/Opti-MEM mix. Briefly, dicer-substrate short interfering RNA (dsiRNA) was complexed with Lipofectamine RNAiMax (Cat. No. 13778075, Thermo) at room temperature for 15 min according to manufacturer’s specifications and the transfection mix was added to the cells at a final concentration of 20 to 30 nM dsRNA/1.0 × 10^6^ cells for 48 h before treatments were performed.

### Protein extraction and immunoblotting

Cells were washed with ice-cold PBS (3×) and lysed in RIPA buffer (contains 150 mM NaCl, 1.0% IGEPAL CA-630, 0.5% sodium deoxycholate, 0.1% SDS, 50 mM Tris, pH 8.0, Cat. No. R0278, Sigma) supplemented with 2 μM TMG, 1 mM PMSF and 1× protease and phosphatase inhibitors (cOmplete, mini, EDTA-free and PhosSTOP respectively, Millipore, Sigma). Lysates were sonicated and protein concentration was quantified using the bicinchoninic acid (BCA) assay (Cat. No. 23225, Pierce). For Western blot, protein samples were combined with 50 mM DTT and 1× LDS sample buffer (NP0008, NuPAGE, Thermo), heat-denatured at 65 °C for 10 min, and 10 μg total protein per sample was resolved by gel electrophoresis. In other experiments, the mid-ventricles of flash frozen hearts (30–50 mg) were homogenized in ice-cold RIPA buffer supplemented with 1% SDS (ratio: 20 μl buffer/mg tissue) using a saw-tooth homogenizer (PowerGen, Fisher). Samples were briefly sonicated and then gently spun at 1200×*g*/1 min. If significant precipitation of proteins was observed a second round of sonication/centrifugation was performed. The cleared homogenates were transferred to fresh tubes and a sample volume was diluted 20× for the BCA. All homogenates were standardized at 2.86 mg/ml with homogenization buffer and then mixed with DTT/LDS for a final protein concentration of 2 mg/ml for Western blot. After heat-denaturation at 65 °C for 10 min, 20 μg total protein per sample were resolved by gel electrophoresis.

For gel electrophoresis, we used NuPAGE Bis-Tris 4 to 12% gradient midi gels (Cat. No. WG1403, Thermo). Proteins were transferred onto nitrocellulose membranes using the iBlot2 (Thermo), or the TransBlot Turbo (BioRad) systems. The membranes were blocked with 5% BSA in TBS for 1 h at room temperature and then incubated with primary antibodies, typically at a 1000:1 dilution in 5% BSA in 0.1% Tween20-TBS, overnight at 4 °C (see Table S1 for a list of primary antibodies used). For detection, we used the LI-COR Odyssey system and appropriate host-specific secondary antibodies (*e.g.* IRDye 800w goat anti-rabbit, Cat. No. 926-32211, LI-COR) typically at a 5000:1 dilution in 5% BSA in 0.1% Tween20-TBS. For the detection of biotinylated targets, we used IRDye800w Streptavidin (Cat. No. 926-32230, 5000:1 in % BSA in 0.1% Tween20-TBS). Quantification of band intensities was performed with Image Studio Lite (LI-COR). Normalization for protein loading was performed based on intensities obtained by staining the membranes with REVERT 700 Total Protein Stain (Cat. No. 926-11010, LI-COR).

### Metabolic labeling with L-azidohomoalanine, azide-alkyne click reaction, and detection of newly synthesized proteins

The procedure was developed based on protocols described previously (65). Briefly, for the metabolic labeling of nascent polypeptides with the clickable methionine analog L-azidohomoalanine, cells were incubated in DMEM without L-methionine, L-cystine and L-Glutamine (Cat. No. 21013024, Thermo), supplemented with 25 μM L-azidohomoalanine (Cat. No. 900892, Sigma), with or without other drugs and for the indicated durations as specified in the figure legends. Following metabolic labeling, the cells were washed in ice-cold PBS (3×), lysed in a buffer containing 1% SDS, 2 μM TMG, protease and phosphatase inhibitors, 50 mM Tris HCl pH 8.0, and sonicated as described above. For the labeling of AHA-incorporating proteins with biotin, the copper-catalyzed azide-alkyne reaction contained 20 μg protein suspension in 0.6% SDS, 2 mM sodium ascorbate, 100 μM THPTA, 1 mM CuSO_4_.5H_2_O and 20 μM biotin alkyne (Cat. B10185, Thermo). In some experiments, a negative control click reaction was set up as above but omitting the catalyst Cu/THPTA. The ‘click’ reaction took place for 20 min at room temperature in the dark and was quenched with 5 mM EDTA. The labelled proteins were then cleared from reactants by methanol: chloroform precipitation. Briefly, this was done by mixing one volume sample/3 volumes methanol/0.75 volumes chloroform/2 volumes water, with vortexing after each addition. The solution was then spun at 16,100*g* to obtain a protein disk that was carefully washed with 2.5 volumes methanol three times followed by a gentle drying step at room temperature for 5 min. The protein disk was solubilized in 1% SDS, 20 mM HEPES, pH 7.9 by warming at 55 °C and followed by gentle sonication to fully resolve any insoluble. The protein samples were then mixed with Western blot buffer to a final concentration of 1× LDS (Cat. No. NP008, Thermo), 50 mM DTT and supplemented with protease/phosphatase inhibitors. The samples were heated at 65 °C for 10 min to fully reduce S-S bonds and then resolved on 4 to 12% Bis-Tris gradient gels for Western blot transfer. Biotinylated proteins were detected with IRDye800w streptavidin and their signal was expressed relative to total protein content obtained by staining for total protein as described in the previous section.

### RNA isolation, reverse transcription and gene expression analysis by quantitative real-time PCR

Total RNA was extracted from NRVMs seeded at 1.0 ×10^6^ cells/well after appropriate treatments as indicated in the main text. The treated cells were washed with ice-cold PBS (3×) and RNA was extracted with the RNeasy kit (Cat. No. 74704, Qiagen), including a proteinase K digestion step to remove abundant sarcomeric proteins. In-column DNaseI treatment (Cat. No. 79254, Qiagen) was used to digest genomic DNA. For reverse transcription (RT), we used a QuantiTect reverse transcription kit (Cat. No. 205311, Qiagen) and an input of 850 ng of total RNA. To control for potentially residual genomic DNA carry-over, reactions without reverse-transcriptase (No-RT) were set up in parallel. Real-time quantitative PCR was performed with the Power SYBR Green master mix (Cat. No. 4367659, Thermo) in the presence of 500 nM forward and reverse primers (for gene-specific sequences see Table S2). A total of 45 cycles of amplification were done (15 s at 95 °C for melting followed by 30 s at 60 °C for annealing and elongation) using the CFX384 Touch system (BioRad). Each sample was run in technical triplicates. Gene expression was quantified with the ΔC_t_ method using B2m as housekeeping gene.

For cardiac gene expression analysis, RNA was isolated from mouse heart samples using TRIzol reagent (Cat. No. 15596026 Invitrogen). Briefly, the heart tissue was homogenized in TRIzol (1 ml per 50 mg tissue) using a bead mill tissue homogenizer (Retch). The subsequent steps for phase separation, RNA precipitation with isopropanol, and washes with ethanol were performed essentially according to the manufacturer’s protocol. The cleared RNA was gently dried for 10 min at room temperature and was subsequently resuspended in RNase free water and measured by a Nanodrop spectrophotometer. Target transcripts were quantified using One-Step RT-qPCR Kit (Cat. No. E3005, New England Biolabs) using an input of 150 ng RNA in the presence of 500 nM forward and reverse primers (for primer sequences see Table S2). Each biological sample was run as technical duplicates and reactions without reverse-transcriptase (No-RT) were set up in parallel to check for potential amplification of genomic DNA. The protocol for reverse transcription and amplification was performed on QuantStudio five system (Thermo). The conditions included 10 min at 55 °C (reverse transcription for cDNA synthesis), followed by an inactivation/denaturation step at 95 °C for 1 min. This was followed by 40 cycles of amplification (15 s at 95 °C for melting followed by 30 s at 60 °C for annealing and elongation) using the. A melt curve assessment was performed at the end to assess for potential primer dimers. Gene expression was quantified using the ΔC_t_ method using B2m as housekeeping gene.

### Tamoxifen injection for inducible cardiomyocyte-specific OGT knockout, echocardiography and osmotic pump implantations

Tamoxifen was initially dissolved in DMSO at final concentration of 20 mg/ml by heating and vortexing on a Thermomix. The solution was then mixed with PEG200 (Cat. No. P3015, Sigma), followed by addition of Tween 80, and sterile PBS yielding a final solution of 2 mg/ml tamoxifen, in 10% DMSO, 20% PEG200, 1.0% Tween 80 and 69% PBS. The mice were weighted, and the solution was injected intraperitoneally (IP) using single use tuberculin syringes (29G, 0.5 cc) at a volume 10-times the body weight for a final dosage of 20 mg tamoxifen/kg body weight. The IP injections were repeated daily over the course of four consecutive days and the overall condition of the mice was carefully monitored for any adverse effects.

Transthoracic echocardiography was performed on conscious mice using a Vevo2100 Imaging System (VisualSonics) equipped with a 40-MHz linear array transducer (MS-400-0152). The hair was removed from the chest using nair cream 24 h prior to the echocardiography. For imaging, mice were gently restrained by grasping the scruff of the neck. Pre-warmed ultrasound gel was applied to the chest, and the transducer was positioned to obtain parasternal long-axis and short-axis views. M-mode images were acquired from the parasternal short-axis view at the level of the papillary muscles. At least three image sequences were recorded for each mouse. Left ventricular internal dimensions at end-diastole (LVID;d) and end-systole (LVID;s) were measured from M-mode tracings. Using these values, the LV volumes in diastole and systole were calculated based on the formulas $LV\;Vol;d=\left(\frac{7.0}{2.4+LVID;d}\right)\times LVID;d^{3}$ and $LV\;Vol;s=\left(\frac{7.0}{2.4+LVID;s}\right)\times LVID;s^{3}$ and then the ejection fraction (EF) was calculated according to the formula $EF=100\times \left(\frac{LV\;Vol;d-LV\;Vol;s}{LV\;Vol;d}\right)$. All measurements were performed by a single blinded observer using the Vevo LAB software (VisualSonics).

Alzet osmotic pumps (Model 2004, Cat. No. NC0683145) were used for continuous drug delivery. ISRIB was dissolved in DMSO at a concentration of 11 mg/ml and was subsequently mixed with PEG200 for a final solution of 2.84 mg/ml ISRIB in 50% DMSO and 50% PEG200. Pumps were filled with ISRIB and primed overnight in sterile saline at 37 °C, according to the manufacturer's instructions. Prior to surgery, mice ∼ 30 g body weight, were anesthetized with isoflurane (2–3% for induction, 1–2% for maintenance) and the dorsal area between the scapulae was shaved and disinfected with betadine and 70% ethanol. A small incision (approximately 1 cm) was made in the mid-scapular region of the back and a pocket was created by spreading the subcutaneous connective tissue. The osmotic pump was inserted into the pocket, with the delivery port first and the incision was closed with surgical clips. Pumps were implanted for 28 days delivering approximately 5.28 μl drug/day. After the completion of the 4 weeks, the mice were anesthetized as above, and the old pumps were surgically removed to be replaced with new pumps containing a fresh solution of ISRIB.

### Analysis of amino acids by LC-MS/MS

NRVMs were treated with vehicle (DMSO) or 25 μM OSMI-1 for 6 h. After treatment, the cells (2.5 × 10^6^ per plate) were washed with ice-cold PBS and the dry cell monolayer was exposed to liquid nitrogen vapors and harvested in frozen state. A fraction of the cell pellet was retained for total protein estimation by BCA. The targeted metabolomic analysis of amino acids was performed by the Metabolomics Core Facility at the University of Pennsylvania. Briefly, small molecules and metabolites were extracted in methanol:water (80:20, v/v) and dried under nitrogen gas and reconstituted in mobile phase. Amino acids were separated and quantified using liquid chromatography-mass spectrometry (LC-MS) on an Agilent 1290/6495 triple quadrupole mass spectrometer. Chromatographic separation was achieved on a C18 column using a gradient of water and acetonitrile with 0.1% formic acid. Multiple reaction monitoring (MRM) transitions specific to each amino acid were used for detection and quantification. Amino acid concentrations were determined using calibration curves generated from stable isotope-labeled internal standards with known concentrations. The quantity of each amino acid in the samples was calculated in nmoles and was expressed relative to mg of total protein in the starting material.

### Statistics

Data were statistically evaluated and plotted using GraphPad Prism. Values in the graphs are reported as means ± standard error. Replicate counts are printed as numerals on each bar, and dots denote individual biological replicates (independently prepared wells, plates, or animals). Data were visually inspected, and where appropriate, outliers were identified using the ROUT method (Robust Regression and Outlier Removal, Q = 1%). Data were examined for normality of distribution using the Shapiro-Wilk test. If the majority of groups within an experiment had normally distributed data, we used the Tukey *post hoc* test. If the distribution of the data in the majority of groups was not normal, we used the Dunnet *post hoc* test. To identify statistically significant differences between two groups we used unpaired Student’s *t* test. To compare three groups, we used one-way ANOVA. To compare four or more groups, we used one-way ANOVA, if the different groups were resulting from drug treatment over time, or from drug combinations. For comparisons between groups resulting from a combination of drug treatment and gene manipulation we used two-way ANOVA. In all cases, the α level for statistical significance was 0.05. *p* values less than 0.05, 0.01, 0.001, and 0.0001 are symbolized as ∗, ∗∗, ∗∗∗, and ∗∗∗∗ respectively. Comprehensive one-way ANOVA results (F statistics, overall *p* values, and all pairwise *post hoc* comparisons) are reported in Table S4. Similarly, the two-way ANOVA results (factor-level *p* values for main effects and interactions, together with adjusted *post hoc* comparisons) are reported in Table S5.

### Study approval

Procedures involving the use of animals were approved by the Institutional Animal Care and Use Committee (IACUC) at Johns Hopkins School of Medicine.

## Data availability

All data presented are included in the article and source data will be made available upon request from the corresponding author.

## Supporting information

This article contains supporting information.

## Conflict of interest

The authors declare that they do not have any conflicts of interest with the content of this article.

## Supporting information

Supporting File 3

Suppl Figure 1

Suppl Figure 2

Suppl Figure 3

Suppl Figure 4

Suppl Figure 5

Suppl Figure 6
