# Supplementary material for: Loss of O-GlcNAcylation in cardiac myocytes triggers the integrated stress response, contributing to heart failure[image]
Source: J Biol Chem. 2025 Oct 14;301(12):110818. doi: 10.1016/j.jbc.2025.110818 (PMC12661449; doi:10.1016/j.jbc.2025.110818)
Supplement: Supporting File 1 [file mmc1.pdf]

Supplemental table 1. Reagent list

REAGENT	SOURCE	IDENTIFIER
**Antibodies**		
**Mouse monoclonal anti-O-GlcNAc**	Zachara Lab	RL2
**Rabbit monoclonal anti- MGEA5/OGA [EPR7154(B)]**	Abcam	Cat# ab124807, RRID:AB_10971848
**Rabbit polyclonal anti- MGEA5/OGA**	Proteintech	Cat# 14711-1-AP, RRID:AB_2143063
**Rabbit monoclonal anti- OGT (D1D8Q)**	Cell Signaling Technology	Cat# 24083, RRID:AB_2716710
**Rabbit polyclonal anti- OGT**	Zachara Lab	AL24
**Rabbit monoclonal anti-phospho-eIF2alpha (Ser51) (D9G8)**	Cell Signaling Technology	Cat# 3398, RRID:AB_2096481
**Rabbit monoclonal anti- eIF2alpha (D7D3)**	Cell Signaling Technology	Cat# 5324, RRID:AB_10692650
**Rabbit monoclonal anti-Ubiquitin (E4I2J)**	Cell Signaling Technology	Cat# 43124, RRID:AB_2799235
**Rabbit anti-ATF4**	Cell Signaling Technology	Cat# 11815, RRID:AB_2616025
**Mouse monoclonal anti-CHOP (L63F7)**	Cell Signaling Technology	Cat# 2895, RRID:AB_2089254
**Rabbit monoclonal anti- Phospho-PERK (Thr980) (16F8)**	Cell Signaling Technology	Cat# 3179, RRID:AB_2095853
**Rabbit monoclonal anti- PERK (C33E10)**	Cell Signaling Technology	Cat# 3192, RRID:AB_2095847
**Rabbit monoclonal anti-Phospho GCN2 (Thr899) (E1V9M) Antibody (Human Preferred)**	Cell Signaling Technology	Cat# 94668
**Rabbit polyclonal anti- GCN2**	Cell Signaling Technology	Cat# 3302, RRID:AB_2277617
**Rabbit polyclonal anti- EIF2AK2, PKR**	Proteintech	Cat# 18244-1-AP, RRID:AB_2246451
**Rabbit monoclonal anti- S6 Ribosomal (5G10) Protein**	Cell Signaling Technology	Cat# 2217 RRID:AB_331355
**Rabbit polyclonal anti- Phospho-S6 Ribosomal Protein (Ser240/244)**	Cell Signaling Technology	Cat# 5364, RRID:AB_10694233
**Rabbit polyclonal anti-Phospho-4E-BP1 (Ser65)**	Cell Signaling Technology	Cat# 9451, RRID:AB_330947
**Rabbit polyclonal anti- 4E-BP1 Antibody**	Cell Signaling Technology	Cat# 9452, RRID:AB_331692
**Rabbit monoclonal anti- mTOR (7C10)**	Cell Signaling Technology	Cat #2983 RRID:AB_2105622
**Rabbit monoclonal anti- Phospho-mTOR (Ser2448) (D9C2)**	Cell Signaling Technology	Cat #5536 RRID:AB_10691552
**Rabbit anti-O-GlcNAc multimAb (mix of monoclonal antibodies)**	Cell Signaling Technology	Cat# 82332, RRID:AB_2799991
**Animal strains**		
**OGT flox mice** (B6.129-Ogttm1Gwh/J)	Jackson Laboratory	Stock No. 004860
**Myh6.MerCreMer transgenic mice** ($A1cf^{Tg(Myh6\text{-}cre/Esr1^{*})1Jmk}$/J)	Jackson Laboratory	Stock No. 005650
**Rosa26.tdTomato.Lox.Stop.Lox reporter mice** B6.Cg-$Gt(ROSA)26Sor^{tm9(CAG\text{-}tdTomato)Hze}$/J or Ai9 (RCL-tdT)	Jackson Laboratory	Stock No. 007909

Chemicals		
**OSMI-1**	Sigma-Aldrich	Cat#SML1621; CAS No.: 1681056-61-0
**Thiamet-G**	Tocris	Cat# 4390; CAS No.: 1009816-48-1
**OSMI-2**	Sigma-Aldrich	Cat#SML2989; CAS No.: 2260542-60-5
**5S-GlcNHex**	MedKoo Biosciences	Cat#466074; CAS No.: 1346636-43-8
**(R)-(−)-Phenylephrine hydrochloride**	Sigma-Aldrich	Cat#P6126; CAS No.: 61-76-7
**MG132**	Sigma-Aldrich	Cat# M8699; CAS No.: 1211877-36-9
**L-Azidohomoalanine hydrochloride**	Sigma-Aldrich	Cat#900892; CAS No.: 942518-29-8
**THPTA (tris-hydroxypropyltriazolylmethylamine)**	Click Chemistry Tools	Cat#1010; CAS No.: 760952-88-3
**Sodium L-Ascorbate**	Sigma-Aldrich	Cat#11140; CAS No.: 134-03-2
**$CuSO_4.5H_2O$**	Sigma-Aldrich	Cat# 469130; CAS No.: 7758-99-8
**Streptavidin IR Dye 800cw**	Li-Cor	Cat# 926-32230
**Torin 2**	MedChemExpress	HY-13002; CAS No.: 1223001-51-1
**ISRIB**	MedChemExpress	HY-12495; CAS No.: 1597403-47-8
**GCN2iB**	MedChemExpress	HY-112654; CAS No.: 2183470-12-2
**GSK2606414**	MedChemExpress	HY-18072; CAS No.: 1337531-36-8
**PKR-IN C16**	MedChemExpress	HY-13977A; CAS No.: 608512-97-6
**$NaAsO_2$**	Sigma-Aldrich	S7400; CAS No.: 7784-46-5
**Poly-I:C**	Sigma-Aldrich	P1530; CAS No.: 42424-50-0
**Halofuginone**	MedChemExpress	HY-N1584; CAS No.: 55837-20-2
**Calyculin A**	Cell Signaling Technology	Cat# 9902; CAS No.: 101932-71-2
**GlutaMAX supplement**	Thermo	Cat# 35050061
**Tamoxifen**	Sigma-Aldrich	T5648; CAS No.:10540-29-1
**Polyethylene glycol (PEG) 200**	Sigma-Aldrich	P3015; CAS No.: 25322-68-3
**Dimethyl sulfoxide**	Sigma-Aldrich	Cat# 472301; CAS No.: 67-68-5
**Osmotic Pumps**	Alzet	Model 2004
**Biotin Alkyne**	Thermo	B10185; CAS. No.: 773888-45-2

Experimental models: Organisms/strains		
**Timed pregnant female rats, outbred (for NRVM isolation)**	Envigo	Hsd:Sprague Dawley Rat

Oligonucleotides		
**ds RNA sequence targeting rat HRI (rn.Ri.Eif2ak1.13.1)**<br>top: GUC AAG AUA AUU CUU ACA UGC AGA A<br>bottom: UUC UGC AUG UAA GAA UUA UCU UGA CAA	IDT DNA	Transcript: NM_013223<br>Targets exon 4
**ds RNA sequence targeting rat PKR (rn.Ri.Eif2ak2.13.1)**<br>top: CAA AUG GAA UUC UGU GAU AAA GGA A<br>bottom: UUC CUU UAU CAC AGA AUU CCA UUU GGA	IDT DNA	Transcript: NM_019335<br>Targets exon 9
**ds RNA sequence targeting rat PERK (rn.Ri.Eif2ak3.13.1)**<br>top: GAU UGG AAG GUC AUG GCG UUU AGT A<br>bottom: UAC UAA ACG CCA UGA CCU UCC AAU CAG	IDT DNA	Transcript: NM_031599<br>Targets exon 5
**ds RNA sequence targeting rat GCN2 (rn.Ri.Eif2ak4.13.1)**<br>top: AUG AAA ACA GUA AAA GUC AGA AUC A<br>bottom: UGA UUC UGA CUU UUA CUG UUU UCA UCU	IDT DNA	Transcript: NM_001105744<br>Targets exons 13,14
**ds RNA sequence targeting rat GCN2 (rn.Ri.Eif2ak4.13.3)**<br>top: AUA AUG GAA UGU UGU GCA UUC AAT C<br>bottom: GAU UGA AUG CAC AAC AUU CCA UUA UUC	IDT DNA	Transcript: NM_001105744<br>Targets exons 39
**ds RNA sequence targeting rat GADD34 (rn.Ri.Ppp1r15a.13.1)**<br>top: GGG GAC UUU CAG AUA UUG AUG AAC A<br>bottom: UGU UCA UCA AUA UCU GAA AGU CCC CAA	IDT DNA	Transcript: NM_133546<br>Targets exon 2
**ds RNA sequence targeting rat CReP (rn.Ri.Ppp1r15b.13.1)**<br>top: GGA UGA GCU UUU CUC ACA AGA ACT G<br>bottom: CAG UUC UUG UGA GAA AAG CUC AUC CUC	IDT DNA	Transcript: NM_001107175<br>Targets exon 2

Software and algorithms		
**Fiji/ImageJ**	Schneider et al., 2012	https://imagej.nih.gov/ij/
**ImageStudio Lite**	LiCor	https://www.licor.com/bio/image-studio-lite/
**Graphpad Prism**	GraphPad Software	www.graphpad.com
