# Supplementary material for: Loss of O-GlcNAcylation in cardiac myocytes triggers the integrated stress response, contributing to heart failure[image]
Source: J Biol Chem. 2025 Oct 14;301(12):110818. doi: 10.1016/j.jbc.2025.110818 (PMC12661449; doi:10.1016/j.jbc.2025.110818)
Supplement: Supporting File 2 [file mmc2.pdf]

Supplemental table 2. Primers used in real-time quantitative PCR (For rat cardiomyocytes or mouse hearts)

Target gene	Species	Forward Sequence (5´ to 3´)	Reverse Sequence (5´ to 3´)	Accession No.
**Atf4**	Rat	GCC ATC TCC CAG AAA GTT TAA TA	GGA ATG CTC TGG AGT GGA AGA C	NM_009716.3
**Chop**		CCA GCA GAG GTC ACA AGC AC	CGC ACT GAC CAC TCT GTT TC	NM_001109986.1
**Atf6**		CGA AGG GAT CAC CTG CTA TTA C	ACT TCA TAA TCC TGC CCA TTG A	NM_001107196.1
**Xbp1**		AGA ACC AGG AGT TAA GGA CAC GC	CCA TGG GAA GAT GTT CTG GG	NM_013842.3
**Xbp1 spliced**		GGT CTG CTG AGT CCG CAG CAG G	GAA AGG GAG GCT GGT AAG GAA C	NM_013842.3
**Dpagt1**		TCA GAT CAT CCC CTG CCC TC	GTG CCC AAG AAA GAG AGG CT	NM_199388.2
**Gfat1**		GAA GCC AAC GCC TGC AAA ATC	CCA ACG GGT ATG AGC TAT TCC	NM_001005879.1
**Gnpnat1**		GTG GCT ACA GCA ACT CTG AT	GGA ACC TCC GAC ACA TGT AA	NM_001134757.1
**Pgm3**		GGA CTG TCG GTT CTG CTG TT	AGC GTC GCT ATC TTG TCT CC	NM_001401193.1
**Uap1**		ACC TGC TGC AGT TCT GGA	CAG CTG GTC TTG ATC TCT GGT	NM_001191930.1
**Glud1**		ACA GCA GAG TTC CAG GAC AG	GTC TAT GTG AAG GTC ACG CC	NM_012570.2
**Gls1**		GAA GGC ACA GAC ATG GTT GGG ATA C	TTA GCC AAG GTT GCA GCC ATC AC	NM_012569.4
**Got2**		ATC GAG CAG GGC ATC AAT GT	AGG CCC ATG TTC TTG GCA T	NM_013177.2
**Hri/Eif2ak1**		GTG CTA CGG GAA GTG AAG GT	GGA ACT CTG TCT TGT GGC TGA	NM_013223.2
**Gcn2/Eif2ak4**		CTG CGG GTC CCT TTT GC	AAT CGG TCT AAC TTC CTA GGT CTG AA	NM_001105744.2
**Gadd34**		ATG GAG TAA AGC AGC CCA GA	CTT GTC CTG GCT TCC CAT TA	NM_133546.3
**Crep**		GCT GGG TGA GGC ACT TTCT	AAC CTG CAT CCA TCC CTT GC	NM_001107175.1
**B2m**		CGA GAC CGA TGT ATA TGC TTG C	GTC CAG ATG ATT CAG AGC TCC A	NM_012512.2
**OGT**	Mouse	GCT TAC TTG TCT AGG ATG TCT CG	TGT TTC CAG ACT TTG CCA CGA	NM_139144.4
**Myh6**		CGA GCT GGA TGA GGC GGA G	TCT GCT GGA GAG GTT ATT CCT CG	NM_001164171.1
**Myh7**		CAT GCC AAC CGT ATG GCT G	GTT CCA CGA TGG CGA TGT TC	NM_080728.3
**Nppa**		GAT GGA TTT CAA GAA CCT GCT AGA	CTT CCT CAG TCT GCT CAC TCA	NM_008725.3
**B2m**		CTG CTA CGT AAC ACA GTT CCA CCC	CAT GAT GCT TGA TCA CAT GTC TCG	NM_009735.3
