# Supplementary material for: Loss of O-GlcNAcylation in cardiac myocytes triggers the integrated stress response, contributing to heart failure[image]
Source: J Biol Chem. 2025 Oct 14;301(12):110818. doi: 10.1016/j.jbc.2025.110818 (PMC12661449; doi:10.1016/j.jbc.2025.110818)
Supplement: Supporting File 3 [file mmc3.pdf]

Supplemental table 3. Genotyping primers

Locus	Primer name	Sequence (5´ to 3´)	Product (bp)
OGT^{flox}	2164OGT.For	GCCATCTCTCCAGCCCCACAAACTG	334 in OGT^{WT} , 483 in OGT^{flox}
	21981OGT.Rev	GACGAAGCAGGAGGGGAGAGCAC	
$Myh6.MerCreMer^{Tg}$	3395cre.For	GGCAATTTCGGCTATACGTAACAGGGT	474 in $Myh6.MCM.cre^{Tg}$, absent in Non-Tg
	4468cre.Rev	GATGAGGTTCGCAAGAACCTGATGGA	
$Rosa26^{WT}$	RosaWT.For	GTGCAAGCACGTTTCCGACTTGA	1020 in $Rosa26^{WT}$
	RosaWT.Rev	GACGGGAGAGGTGATAGACACTGA	
$tdTomato^{KI}$	3908tdTom.For	GAGCAAGGGCGAGGAGGTCATCAAA	1457 in $tdTom^{KI}$
	5364tdTom.Rev	GGCCTTGTACGCGTTAAGTGCAACACA A	
$A1cf^{WT}$ (knock-in locus of MCM)	A1cf704.For	GCATTTCTATTGCACACAGCAATCCAGT GTGA	289 in $A1cf^{WT}$ and Het, absent in $A1cf^{KI}$
	A1cf992.Rev	GAGAGGAGCACCTAGGAGGCAAGCA	
