# Supplementary material for: Loss of O-GlcNAcylation in cardiac myocytes triggers the integrated stress response, contributing to heart failure[image]
Source: J Biol Chem. 2025 Oct 14;301(12):110818. doi: 10.1016/j.jbc.2025.110818 (PMC12661449; doi:10.1016/j.jbc.2025.110818)
Supplement: Suppl Figure 1 [file mmc4.pdf]

Loss of O-GlcNAcylation in cardiac myocytes triggers the integrated stress response contributing to heart failure

Kyriakos N. Papanicolaou, Wenxi Zhang, Aidan Dunphy, Justin C. Zhong, Clara Y. Cho, Dan R. Turner, Deepthi Ashok, D. Brian Foster, Brian O'Rourke & Natasha E. Zachara

Supplemental material

Supplemental figures 1-6

Supplemental tables 1-3

Supplemental Figure 1

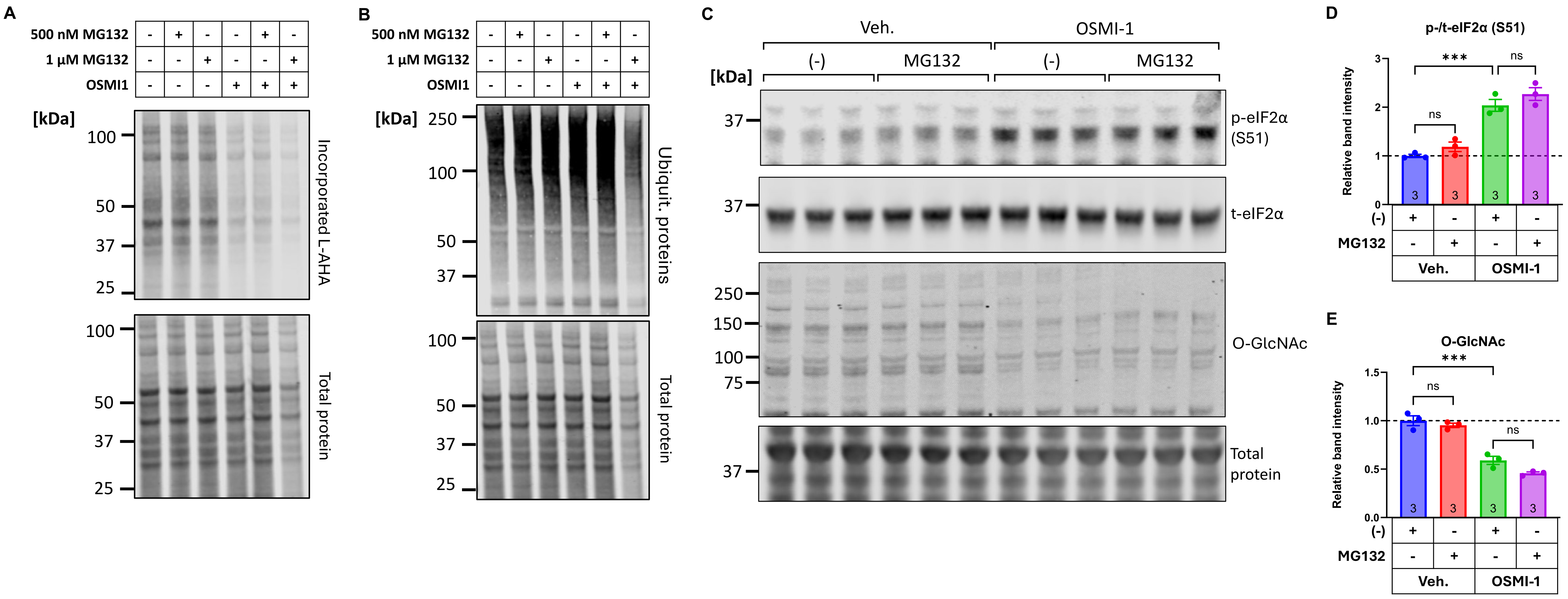

Supplemental Figure 1. Reduction in nascent polypeptide synthesis caused by OGT inhibition is not rescued by proteasome inhibition. (A) NRVMs were exposed to proteasomal inhibitor MG132 (500 nM or 1 μM) with or without OSMI-1 (25 μM) in starvation medium, free from L-methionine and L-cysteine and supplemented with L-AHA (25 μM). After 6 hours of treatment, cells were harvested and subjected to L-AHA-azide biotin-alkyne 'click' reaction. L-AHA incorporation was assessed by western blotting. **(B)** Samples from (A) were blotted for protein-Ubiquitin conjugates to assess the accumulation of ubiquitinated proteins in the presence of MG132 with or without OSMI-1. **(C)** Western blot analysis of eIF2α phosphorylation and overall protein O-GlcNAcylation after treatment for 6 hours with OSMI-1 (25 μM), with or without MG132 (500 nM). **(D-E)** Bar graphs show band densitometry results. Comparisons were performed using one-way ANOVA followed by Tukey's post-hoc test. ns: not significant, * $P < 0.05$, ** $P < 0.01$, *** $P < 0.001$, **** $P < 0.0001$. Complete ANOVA statistics are reported in Supplemental Table 4.
