# Supplementary material for: Loss of O-GlcNAcylation in cardiac myocytes triggers the integrated stress response, contributing to heart failure[image]
Source: J Biol Chem. 2025 Oct 14;301(12):110818. doi: 10.1016/j.jbc.2025.110818 (PMC12661449; doi:10.1016/j.jbc.2025.110818)
Supplement: Suppl Figure 2 [file mmc5.pdf]

Supplemental Figure 2

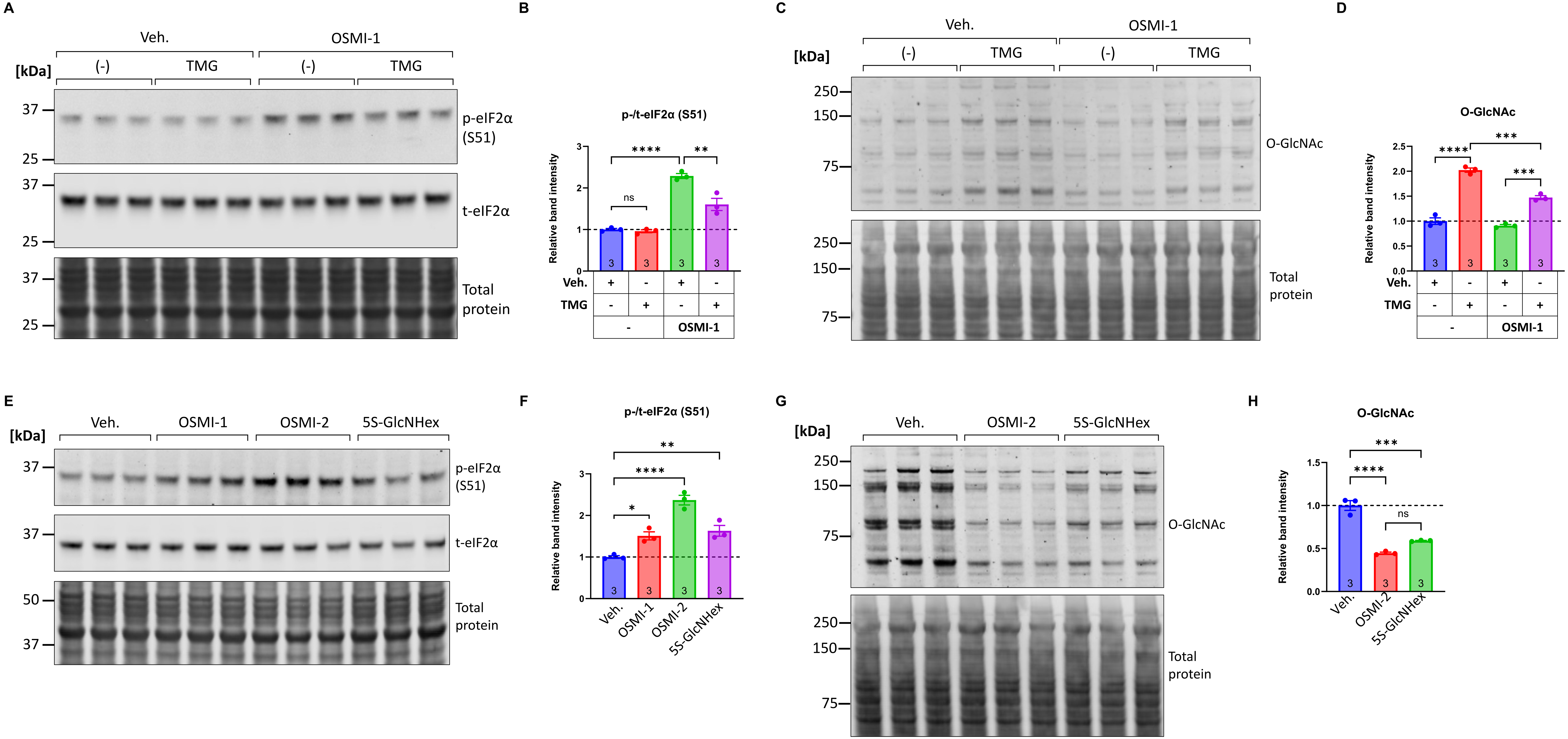

Supplemental Figure 2. Modulation of O-GlcNAc cycling demonstrates consistent regulation of eIF2α phosphorylation. (A-B) NRVMs were treated with or without the OGA inhibitor TMG (25 μM, 3h) followed by treatment with OSMI-1 (25 μM, 3h) or the respective vehicle control (0.1% DMSO). Cells were subsequently lysed and immunoblots performed to assess the relative phosphorylation levels of eIF2α at S51. The results of densitometry quantification are shown in panel B. **(C-D)** Western blot analysis of overall protein O-GlcNAcylation, accompanied by densitometry analysis in the four treatment groups prepared as described in A. **(E-F)** Western blot analysis of phosphorylated and total eIF2α in NRVMs treated with vehicle, OSMI-1, OSMI-2 and 5S-GlcNHex (the final concentration for all inhibitors was 25 μM for 6 h. The results of the densitometry analysis that reports on the relative phosphorylation levels of eIF2α are shown in F. **(G-H)** Western blot analysis of overall protein O-GlcNAcylation after treatment with vehicle control, OSMI-2, and 5S-GlcNHex. The analysis of overall band intensities representing O-GlcNAc levels in each group are shown in panel H. Comparisons across groups were performed using one-way ANOVA with Tukey's post-hoc test. ns: not significant ($P > 0.05$), * $P < 0.05$, ** $P < 0.01$, *** $P < 0.001$, **** $P < 0.0001$. Complete ANOVA statistics are reported in Supplemental Table 4.
