# Supplementary material for: Loss of O-GlcNAcylation in cardiac myocytes triggers the integrated stress response, contributing to heart failure[image]
Source: J Biol Chem. 2025 Oct 14;301(12):110818. doi: 10.1016/j.jbc.2025.110818 (PMC12661449; doi:10.1016/j.jbc.2025.110818)
Supplement: Suppl Figure 3 [file mmc6.pdf]

Supplemental Figure 3

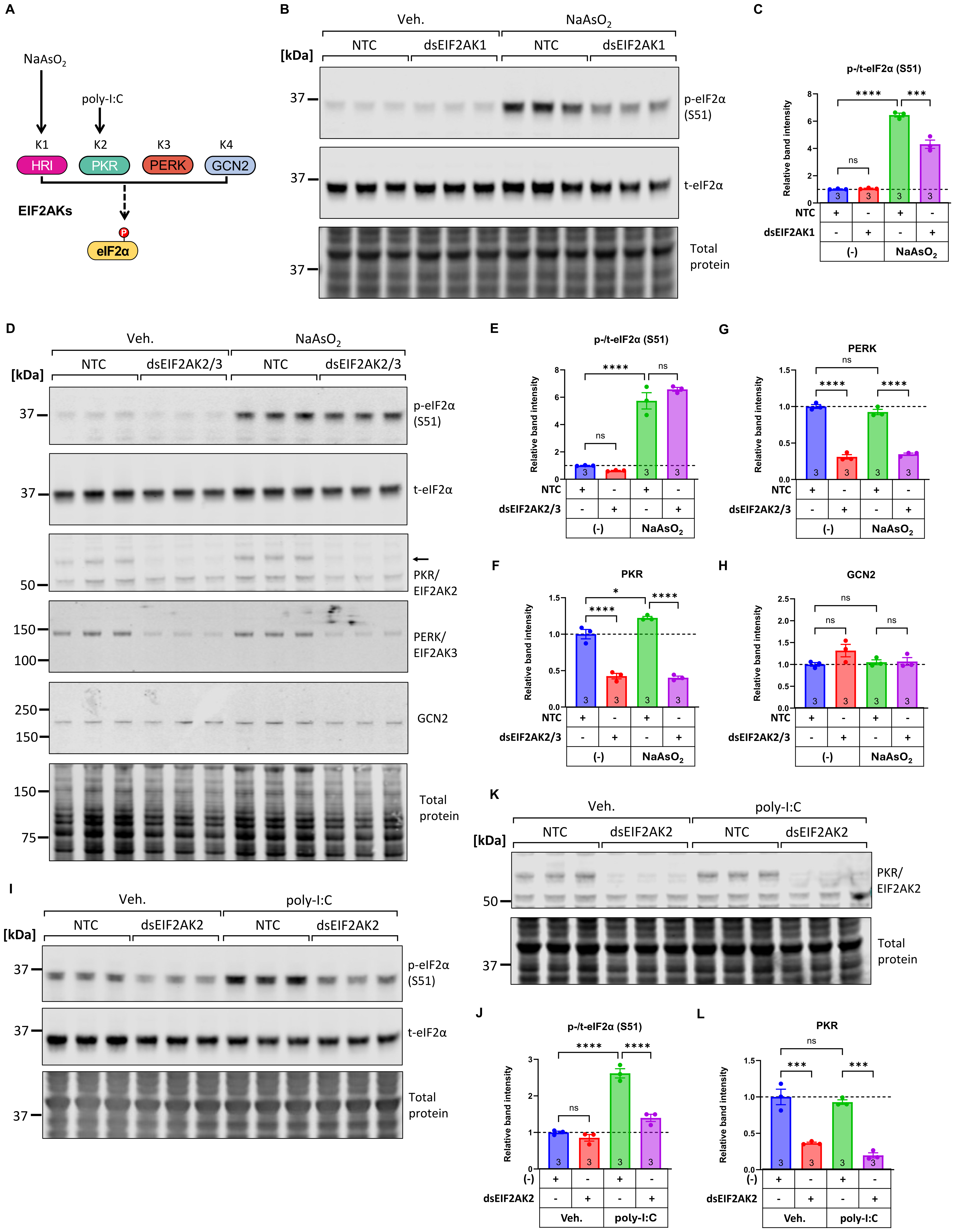

Supplemental Figure 3. Validation of functional knockdown of EIF2AKs HRI and PKR using specific activators sodium arsenite and poly-I:C respectively. (A) Schematic depicting the four eIF2α kinases: HRI (EIF2AK1), PKR (EIF2AK2), PERK (EIF2AK3), and GCN2 (EIF2AK4). The specific activators for the first two kinases are shown: sodium arsenite ($NaAsO_2$), which induces oxidative stress and activates the eIF2α pathway, and poly-I:C, a synthetic analog of double-stranded RNA that activates PKR. **(B-C)** NRVMs were transfected with 20 nM non-targeting control (NTC) dsRNA or dsRNA targeting rat HRI/EIF2AK1. After 24 hours, the media was changed to medium without serum for an additional 24 hours. The cells were then treated with or without sodium arsenite ($NaAsO_2$) at a concentration of 500 µM for 6 hours, and samples were collected for western blot analysis to assess the levels of phospho-eIF2α and total eIF2α. **(D-H)** NRVMs were transfected with 20 nM dsRNA targeting rat EIF2AK2 and EIF2AK3 (20 nM each) or 40 nM non-targeting control (NTC) dsRNA. After 24 hours, the media was changed to medium without serum for an additional 24 hours. The cells were then treated with or without sodium arsenite ($NaAsO_2$) at a concentration of 500 µM for 6 hours, and samples were collected for western blot analysis to assess the levels of phospho-eIF2α, total eIF2α, PERK, PKR, and GCN2. **(I-L)** NRVMs were transfected with 20 nM dsRNA targeting rat EIF2AK2/PKR or 20 nM non-targeting control (NTC) dsRNA. After 24 hours, the media was changed to medium without serum for an additional 24 hours. The cells were then transfected with or without poly-I:C at a concentration of 20 µg/ml, complexed with Lipofectamine RNAiMax. Samples were collected 6 hours later for western blot analysis to assess the levels of phospho-eIF2α, total eIF2α, and PKR. Comparisons across groups were performed using two-way ANOVA followed by Tukey's post hoc test. ns: not significant, * $P < 0.05$, ** $P < 0.01$, *** $P < 0.001$, **** $P < 0.0001$. Complete ANOVA statistics are reported in Supplemental Table 5.
