# Supplementary material for: Loss of O-GlcNAcylation in cardiac myocytes triggers the integrated stress response, contributing to heart failure[image]
Source: J Biol Chem. 2025 Oct 14;301(12):110818. doi: 10.1016/j.jbc.2025.110818 (PMC12661449; doi:10.1016/j.jbc.2025.110818)
Supplement: Suppl Figure 4 [file mmc7.pdf]

Supplemental Figure 4

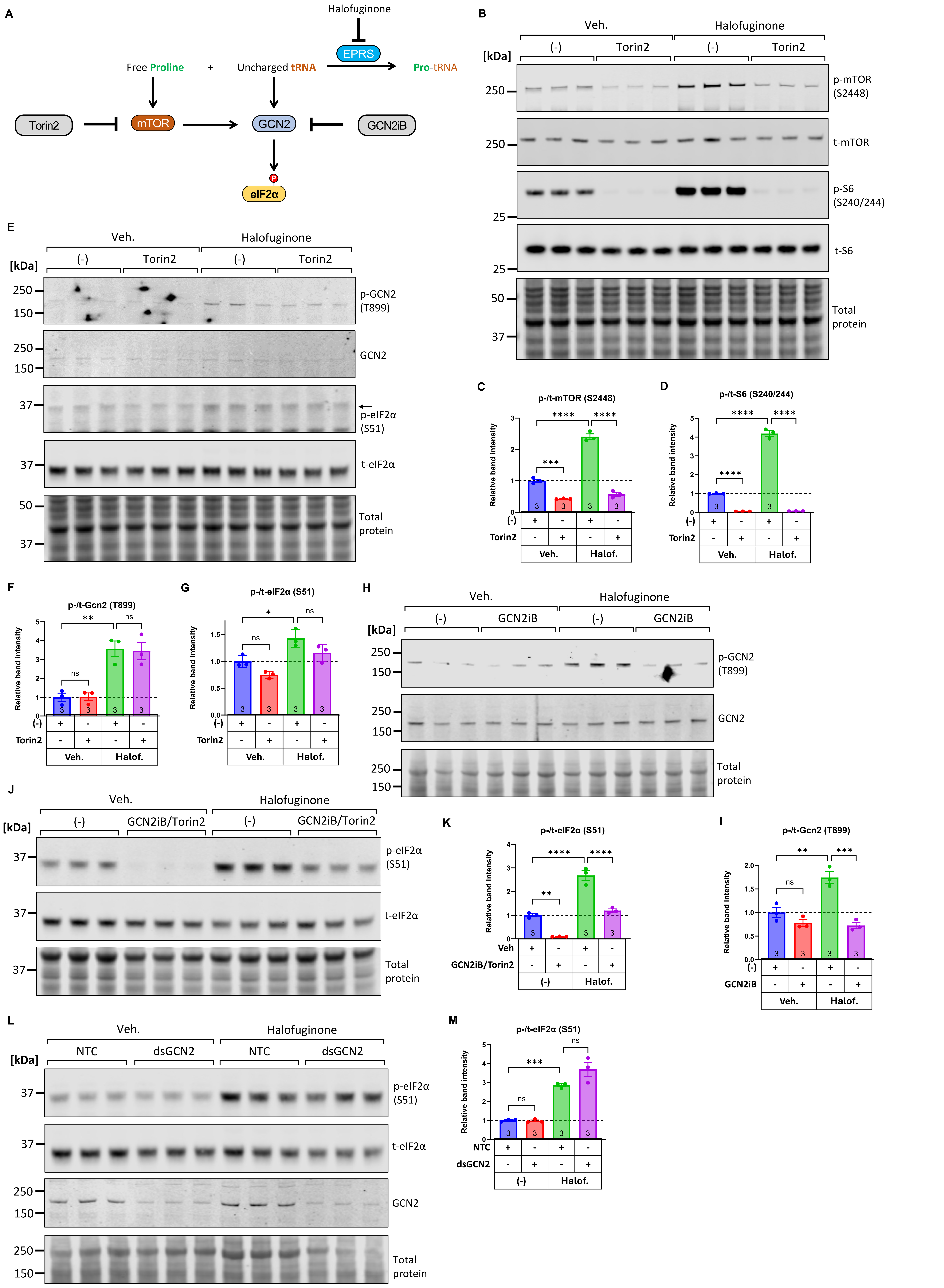

Supplemental Figure 4. Treatment with the tRNA aminoacyl transferase inhibitor halofuginone activates both GCN2 and mTOR, mimicking the effects of OGT inhibition. (A) Proposed model illustrating the effects of mTOR and GCN2 through the impairment of tRNA aminoacylation. The enzyme glutamyl-prolyl-tRNA synthetase (EPRS) is inhibited by halofuginone, a natural product that disrupts the charging of proline to its cognate tRNA, thereby mimicking amino acid limitation. As a result, accumulating free proline activates mTOR, while accumulating uncharged proline tRNA activates GCN2. Activation of both pathways can contribute to eIF2α phosphorylation. **(B-D)** NRVMs were treated with or without Torin2 (1 μM) and with or without Halofuginone (250 nM) for 6 hours. Samples were collected for western blot analysis to assess the levels of phospho-mTOR, total mTOR, phospho-S6, and total S6. **(E-G)** NRVMs were treated with or without halofuginone (250 nM) and with or without Torin2 (1 μM) for 6 hours. Samples were collected for western blot analysis to assess the levels of phospho-GCN2, total GCN2, phospho-eIF2α, and total eIF2α. **(H-I)** NRVMs were treated with or without GCN2iB (10 μM) and with or without halofuginone (250 nM) for 6 hours. Samples were collected for western blot analysis to assess the levels of phospho-GCN2 and total GCN2. **(J-K)** NRVMs co-treated with or without a combination of Torin2 and GCN2iB (1 μM and 10 μM respectively) were exposed to vehicle or Halofuginone (250 nM) for 6 hrs. The samples were analyzed by western blot to assess the levels of phospho-eIF2α, and total eIF2α. **(L-M)** NRVMs were transfected with 30 nM NTC, or 30 nM GCN2-targeting dsRNA. After 48 hours of transfection, cells were treated with or without halofuginone (250 nM) for 6 hours. Samples were collected for western blot analysis to assess the levels of phospho-eIF2α and total eIF2α. Comparisons among groups resulting from drug treatment combinations were performed using one-way ANOVA, while those resulting from a combination of a drug and gene knockdown were performed using two-way ANOVA. In either case we used Tukey's post-hoc test. ns: not significant, * P < 0.05, ** P < 0.01, *** P < 0.001, **** P < 0.0001. Complete ANOVA statistics are reported in Supplemental Tables 4 and 5.
