# Supplementary material for: Loss of O-GlcNAcylation in cardiac myocytes triggers the integrated stress response, contributing to heart failure[image]
Source: J Biol Chem. 2025 Oct 14;301(12):110818. doi: 10.1016/j.jbc.2025.110818 (PMC12661449; doi:10.1016/j.jbc.2025.110818)
Supplement: Suppl Figure 5 [file mmc8.pdf]

Supplemental Figure 5

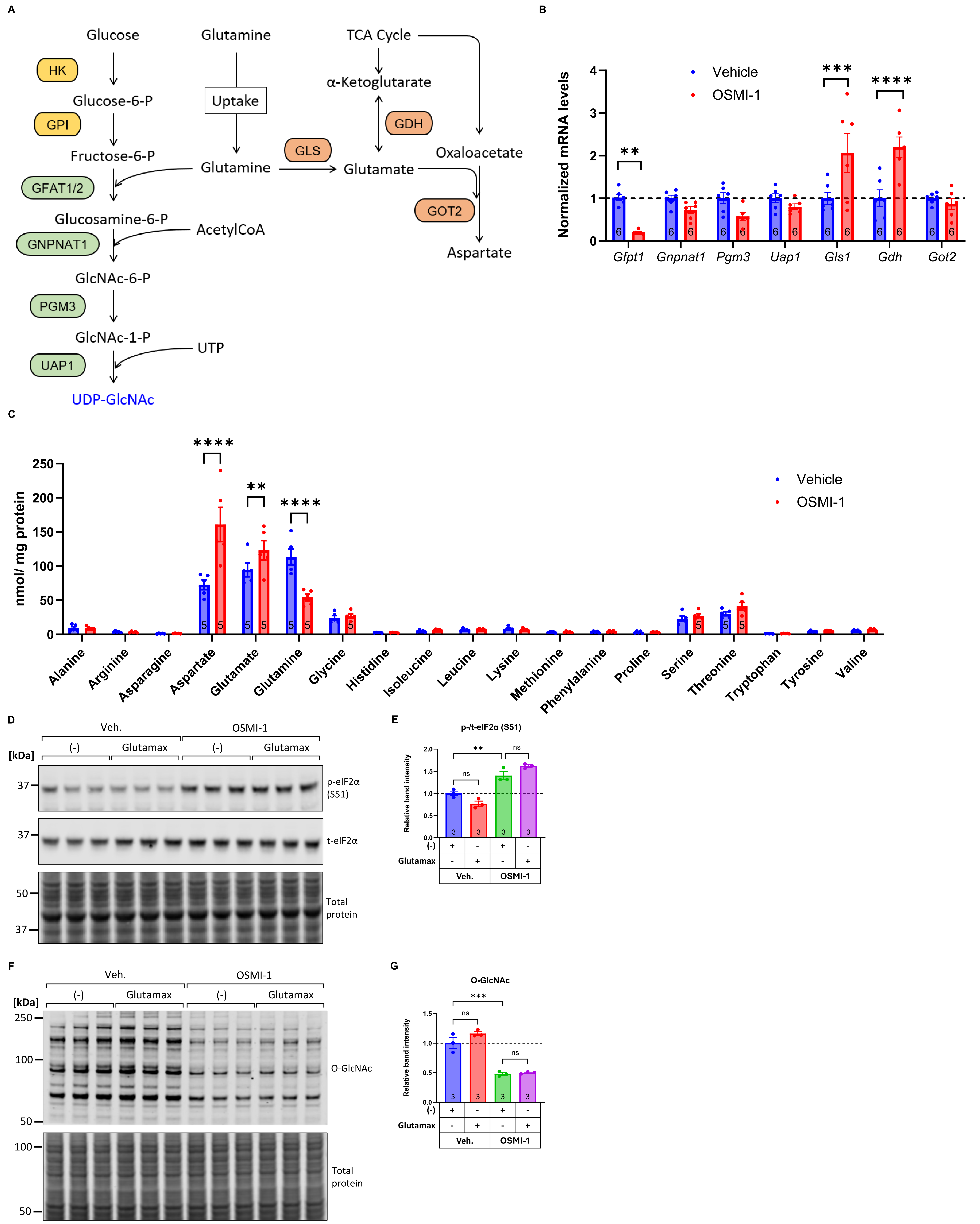

Supplemental Figure 5. OGT inhibition perturbs the expression of metabolic genes involved in amino acid utilization and causes abnormal levels of the amino acids aspartate and glutamine. (A) Schematic representation of the Hexosamine Biosynthetic Pathway (HBP) and related metabolic pathways. Hexokinase (HK), and Glucose-6-phosphate isomerase (GPI) supply the HBP with Glucose-6-Phosphate, the substrate of the HBP rate-limiting enzyme Glutamine:fructose-6-phosphate amidotransferase 1/2 (GFAT 1/2). The HBP also includes the Glucosamine-phosphate N-acetyltransferase 1 (GNPNAT1), Phosphoglucomutase 3 (PGM3), and UDP-N-acetylglucosamine pyrophosphorylase 1 (UAP1). The schematic also shows the interconnected pathways for the biosynthesis of glutamine, glutamate and aspartate and the participation of the Tricarboxylic Acid (TCA) cycle intermediates α-ketoglutarate and oxaloacetate. The key enzymes for these interconversions include Glutaminase (GLS), Glutamine Synthetase (GS), Glutamate Dehydrogenase (GDH) and Glutamate Oxaloacetate Transaminase 2 (GOT2). **(B)** Quantitative real-time PCR analysis of mRNA from cells treated with DMSO or OSMI-1 (25 μM) for 24 hours. The mRNA expression levels of the following genes were measured: *Gfpt1* (the gene encoding for Gfat1), *Gnpnat1*, *Pgm3*, *Uap1*, *Gls1*, *Gdh* and *Got2*. **(C)** Targeted metabolomics analysis was performed on NRVMs treated with DMSO or 25 μM OSMI-1 for 6 hours. 2.5 million cells per sample were harvested in liquid nitrogen and metabolites were extracted in methanol:water (80:20, v/v). Analysis was conducted using liquid chromatography-mass spectrometry (LC-MS) on an Agilent 1290/6495 triple quadrupole mass spectrometer. Metabolites were quantified using isotopically-labeled internal standards. Results are means from five replicates per group and reported as nmol amino acid normalized to mg of protein. **(D-G)** Western blot analysis of NRVMs treated with or without glutamine supplementation (Glutamax) and with or without OSMI-1 (25 μM) for 6 hours. The phosphorylation of eIF2α and the overall O-GlcNAc levels were quantified. Comparisons between vehicle and OSMI-1 groups (Panels B and C) were performed using unpaired t-tests and the P value was adjusted for multiple comparisons using the Holm-Sidak method. Comparisons in Panels E and G were performed using one-way ANOVA with Tukey's post-hoc test. ns: not significant, * $P < 0.05$, ** $P < 0.01$, *** $P < 0.001$, **** $P < 0.0001$. Complete ANOVA statistics are reported in Supplemental Table 4.
