# Supplementary material for: Loss of O-GlcNAcylation in cardiac myocytes triggers the integrated stress response, contributing to heart failure[image]
Source: J Biol Chem. 2025 Oct 14;301(12):110818. doi: 10.1016/j.jbc.2025.110818 (PMC12661449; doi:10.1016/j.jbc.2025.110818)
Supplement: Suppl Figure 6 [file mmc9.pdf]

Supplemental Figure 6

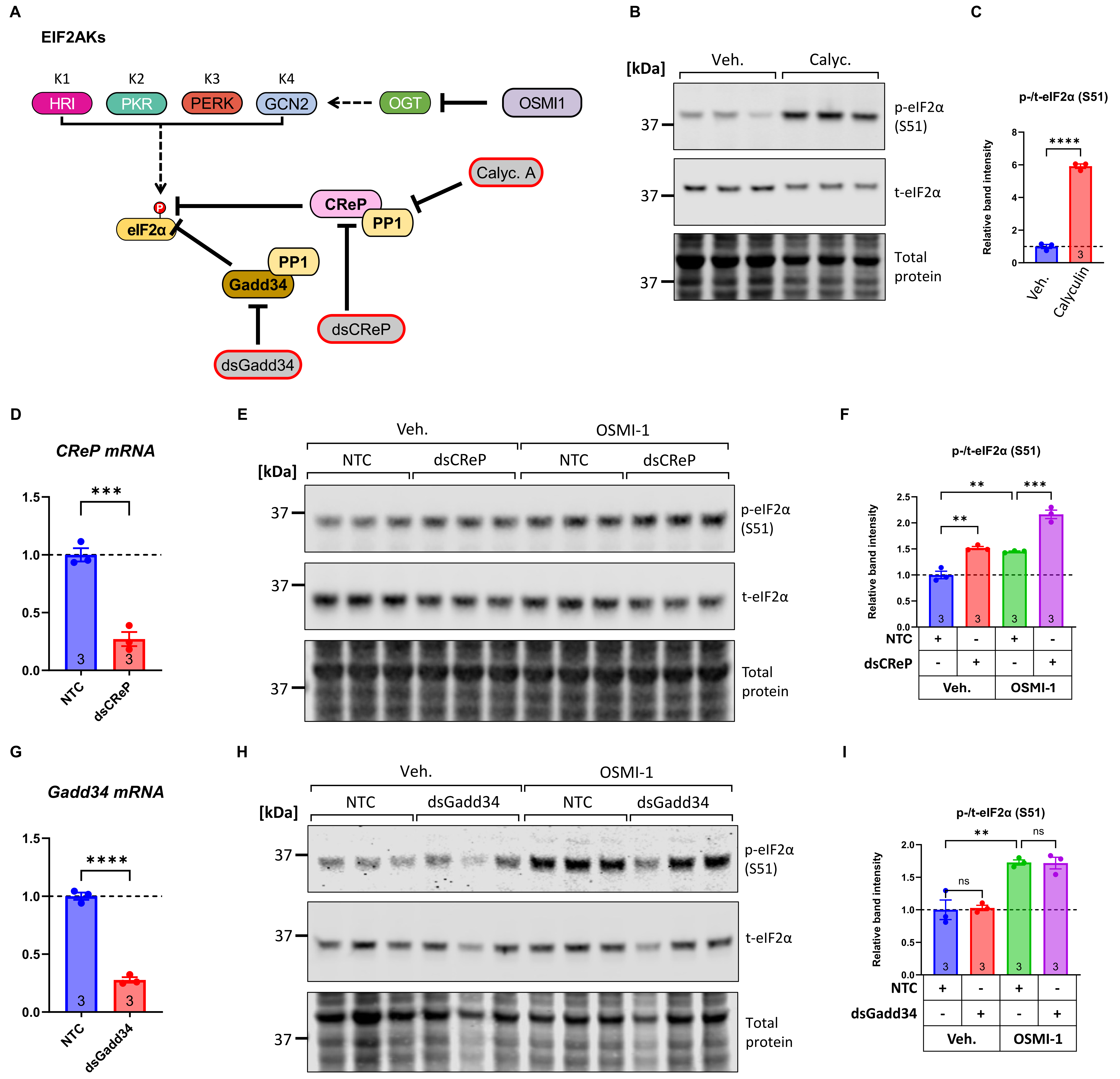

Supplemental Figure 6. Increased eIF2α phosphorylation during OGT inhibition is not attributable to eIF2α phosphatase effects. (A) Schematic representation depicting the regulation of eIF2α phosphorylation and dephosphorylation. The four eIF2α kinases (HRI, PKR, PERK, and GCN2) are shown upstream of phospho-eIF2α. The schematic also illustrates the phosphatase complex composed of Constitutive Repressor of eIF2α phosphorylation (CReP), or Growth Arrest and DNA Damage-Inducible Protein 34 (Gadd34) and Protein Phosphatase 1 (PP1), which dephosphorylates eIF2α. CReP and Gadd34 function as scaffolds for the recruitment of PP1 to eIF2α. Inhibition of this phosphatase complex occurs through Calyculin A, or siRNA targeting CReP or GADD34. **(B-C)** NRVMs were treated with or without Calyculin A (20 nM) for 1 hour. Samples were collected for western blot analysis of phospho-eIF2α and total eIF2α. **(D)** NRVMs were transfected with 20 nM non-targeting control (NTC) dsRNA, or dsRNA targeting rat CReP. After 48 hours, cells were processed for RNA extraction and qPCR to assess *CReP* mRNA levels. **(E-F)** Cells were transfected as in D and 48 hours later were treated with or without OSMI-1 (25 µM) for 6 hours and processed for western blot analysis of phospho-eIF2α and total eIF2α. **(G)** NRVMs were transfected with 20 nM NTC or Gadd34-targeting dsRNA and 48 hours later, they were processed for RNA extraction and qPCR to assess *Gadd34* mRNA levels. **(H-I)** NRVMs were transfected as in G and after 48 hours they were treated with or without OSMI-1 (25 µM) for 6 hours. Samples were collected for western blot analysis of phospho-eIF2α and total eIF2α. Comparisons in C, D and G used unpaired Student's t-tests. In panels F and I, comparisons were performed using two-way ANOVA and Tukey's post-hoc test. ns: not significant, ** P < 0.01, *** P < 0.001, **** P < 0.0001. Complete ANOVA statistics are reported in Supplemental Table 5.
